# A topological characterization of stabilizing consensus

**DOI:** 10.1007/s00446-026-00508-z

**Published:** 2026-06-22

**Authors:** Ulrich Schmid, Stephan Felber, Hugo Rincon Galeana

**Affiliations:** 1 Embedded Computing Systems Group E191-02, TU Wien, Treitlstrasse 1–3, 1040 Vienna, Austria; 2 Internet Architecture and Management, TU Berlin, Einsteinufer 17, 10587 Berlin, Germany

**Keywords:** Distributed computing, point-set topology, stabilizing consensus, impossibility results

## Abstract

We provide a complete characterization of the solvability/impossibility of deterministic stabilizing consensus in virtually any computing model with benign process and communication faults using point-set topology. Relying on the topologies for infinite executions introduced by Nowak, Schmid and Winkler (JACM, 2024) for terminating consensus, we show that semi-open decision sets and semi-continuous decision functions as introduced by Levin (AMM, 1963) are the appropriate means for this characterization: Unlike the continuous decision functions for terminating consensus, semi-continuous functions do not require the inverse image of an open set to be open and hence allow to map a connected space to a disconnected one. We also show that multi-valued stabilizing consensus with weak and strong validity are equivalent, as is the case for terminating consensus. By applying our results to (variants of) all the known possibilities/impossibilities for stabilizing consensus, we easily provide a topological explanation of these results.

## Introduction

A substantial share of distributed computing research has been devoted to terminating tasks like consensus, where every process is given some input value, and must locally compute some output value and then terminate. Still, there are also distributed computing problems that cannot be described by such terminating tasks. Apart from self-stabilizing distributed algorithms [1], which can recover from arbitrarily corrupted states, there are also tasks where the processes are allowed to continuously update their output values. Canonical examples are asymptotic consensus [2, 3], stabilizing consensus [4, 5], and approximate agreement [6, 7]. A number of services in practical distributed systems, like clock synchronization [8, 9] and sensor fusion [10], can be built atop of such non-terminating tasks.

Unlike asymptotic consensus [2, 3, 11–14] and approximate agreement [2, 3, 6, 7, 15–21], which have been studied in various computing models and are hence fairly well-understood, not much is known about stabilizing consensus [4, 5, 22, 23]. In stabilizing consensus, processes need to agree on a common decision value only eventually and do not have to decide irrevocably, i.e., may change their decisions arbitrarily before eventually stabilizing to the common value. As a straightforward relaxation of terminating consensus, stabilizing consensus is of particular interest also for the theory of distributed computing, namely, for studying the solution power of distributed computing models. For example, whereas terminating deterministic consensus is impossible to solve in the synchronous lossy-link model [24–27] or in asynchronous systems in the presence of just a single crash fault [28], deterministic stabilizing consensus can be solved in those models.

More specifically, stabilizing consensus protocols (= algorithms) for asynchronous systems with fair-lossy links were given in [4], both for crash faults and byzantine process faults. Stabilizing consensus protocols for synchronous dynamic networks controlled by message adversaries [29] were proposed in [5], where Charron-Bost and Moran introduced the strikingly simple anonymous MinMax algorithms. In particular, as argued in [23], a simple MinMax algorithm can be used to solve stabilizing consensus in the *lossy-link model* (LL) [24–27], where two processes are connected by a a pair of directed links that may lose at most one message in every synchronous round. In [22], Schmid and Schwarz developed a stabilizing consensus protocol for processes with unique ids for the vertex-stable root message adversary studied in [30], by stripping-off the termination code from a terminating consensus protocol.

Even less is known regarding impossibility results for stabilizing consensus. In [4], it was shown that stabilizing consensus is impossibe to solve in any computing model with byzantine faults and anonymous processes. In [5], a partitioning argument was used to prove that the problem cannot be solved deterministically in synchronous systems controlled by a message adversary if the latter does not guarantee a non-empty kernel, i.e., at least one process that can reach all other processes (possibly via multiple hops) infinitely often. The first non-obvious impossibility result for deterministic stabilizing consensus has been established recently by Felber and Rincon Galeana in [23]. The authors showed that the problem cannot be solved in the *delayed lossy-link model* (DLL), where the links between the two processes may also lose both messages in a round, provided this happens at most *finitely* often. Note that this result negatively answered the question raised in [5], namely, whether a non-empty kernel alone is also *sufficient* for solving stabilizing consensus.

In this paper, we provide a complete characterization of the solvability/impossibility of deterministic stabilizing consensus, in virtually any model of computation with benign process and communication faults, using point-set topology as introduced by Alpern and Schneider in [31]. In more detail:

1. Relying on the topologies for infinite executions introduced by Nowak, Schmid and Winkler [32, 33], we prove that semi-open sets and semi-continuous functions as proposed by Levine in [34] (already^1^ in 1963) are the appropriate means for this characterization. Unlike the decision functions for terminating consensus, which are continuous, they do not require the inverse image of an open set to be open and hence allow to map a connected space to a disconnected one. Since “offending” limit points do not need to be excluded from the set of admissible executions here, this explains why stabilizing consensus is solvable in models where terminating consensus is impossible.
2. We show that, as in the case of terminating consensus, stabilizing consensus with weak and strong validity are equivalent.
3. We demonstrate the power of our novel topological characterization by applying it to (variants of) all the possibility and impossibility results for stabilizing consensus known so far. We also add a new distributed computing model, which we called the *one-message adversary*. Whereas it is deceptively simple, it constitutes something like a “canonical example”, as it “sits” precisely at the border between terminating consensus impossibility and stabilizing consensus possibility.

However, it seems appropriate to add some caveat regarding our topological characterization here. On the positive, our approach is “universally” applicable and translates the operational properties of the stabilizing consensus problem into purely mathematical properties, namely, semi-open sets and semi-continuous functions. It hence admits analysis techniques that are very different from classic ones (more specifically topological theorems), providing insights that are hard or even impossible to obtain via different methods. Among those are the existence of a common broadcaster and the equivalence of strong and weak validity when solving stabilizing consensus, for example.

On the negative side, applying our topological characterization to a non-trivial model requires both familiarity with point-set topology and an intimate understanding of the structure of the model assumptions. This is reasonably easy in the case of possibility results, where one can often focus on the common broadcaster, as in Section 7.1 and Section 7.2, for example, but could be hard for impossibility results like the one of Section 7.2.4. Moreover, we should point out that our generic stabilizing consensus algorithm is not at all “practical”, since it requires full knowledge of the decision sets. Developing practical algorithms will hence very likely require classic algorithm design and analysis techniques anyway, albeit insights from our topological characterization might be beneficial for guiding those, of course.

Our paper is organized as follows: In Section 2, we provide the cornerstones of the generic system model introduced in [32] and the variants of stabilizing consensus considered in this paper. Section 3 briefly recalls some point-set topology basic terms and restates the most relevant results from [34]. Section 4 provides the cornerstones of the topological characterization of terminating consensus [32] needed for our results. In Section 5, we provide our topological characterization for stabilizing consensus, Section 6 is devoted to the equivalence of multi-valued stabilizing consensus with weak and strong validity. Finally, in Section 7, we apply our topological characterization to various possibility/impossibility results. Some conclusions in Section 8 round-off our paper.

## System model

We use the system model introduced in [32], which targets distributed message passing or shared memory systems made up of a set of *n* deterministic processes Π, taken from $[n]=\{1,\dots ,n\}$ for simplicity. Individual processes are denoted by letters *p*, *q*, etc. We assume that all processes are present in an execution already at time t=0, in some initial configuration that also assigns initial values to the processes. In order to also model a process *p* that becomes *active* at some later time, however, we allow *p* to remain *passive* until some time $t^a_p$. While being passive, all computing steps *p* might have to execute (e.g., in a lock-step synchronous computing model) are no-operation steps (that do not change *p*’s state at all), and all messages possibly arriving at *p* are lost without a trace.

We restrict our attention to *full-information executions*, in which processes continuously relay all the information they gathered to all other processes and apply some local decision function in every computing step. The exchanged information includes the process’s initial value, but also, more importantly, a record of all events (message receptions, shared memory readings, object invocations, ...) witnessed by the process. Our general system model is hence applicable whenever no constraints are placed on the size of the local memory and the size of values to be communicated (e.g., message/shared-register size). In particular, it is applicable to classical synchronous and asynchronous message-passing and shared-memory models with benign^2^ process and communication faults, see [32, App. A] for details.

Formally, an execution is a sequence of (full-information) *configurations*, which are the vectors of the processes’ local *views* (= local states). Every configuration represents the processes’ view of a *global system state*, where the latter also contains information that is *not* accessible to the processes, like the internal state of the communication medium. Consult [32, App. A] for an in-depth discussion of the related issues.

For every process $p\in \Pi$, there is an equivalence relation $\sim _p$ on the set $\mathcal {C}$ of all possible configurations—the *p*-indistinguishability relation—indicating whether process *p* can locally distinguish two configurations, i.e., if it has the same view $V_p(C)=V_p(D)$ in *C* and *D*. In this case we write $C\sim _p D$. Note that two configurations that are indistinguishable for all processes need not correspond to the same global system state.

*Executions* are represented by infinite sequences of configurations, which represent the evolution of a distributed computation. We completely abstract away how executions are generated, which is traditionally (see e.g. [35]) modeled by means of a transition function and a message sending function encoding a distributed algorithm, which are performed by a process when it executes a computing step. We just assume that we are given the set $$\Sigma \subseteq \mathcal {C}^\omega $$ of *admissible executions*, where $$\gamma \in \Sigma $$ is just a sequence $$\gamma = (C^t)_{t\ge 0}$$ of configurations. Herein, $$t \in \mathbb {N}_0 = \mathbb {N}\cup \{0\}$$ denotes a notion of *global* time that is not accessible to the processes. $$C^0$$ is the *initial* configuration of γ, and any configuration $$C^{t'}$$ occurring in γ is called *reachable* from any configuration $$C^t$$ in this execution if $t\leq t^{\prime }$. $$\mathcal {C}^\omega $$ is the set of all possible infinite configuration sequences, whereas Σ is the subset of the configuration sequences that are *admissible*, i.e., can occur, in the given computing model.

In addition to the indistinguishability relations, we assume the existence of a function $${{\,\textrm{Ob}\,}}:\mathcal {C}\rightarrow 2^\Pi $$ that specifies the set of *obedient* processes in a given configuration. Obedient processes must follow the protocol and satisfy the task specification, i.e., the purpose of their protocol, as e.g. specified in Definition 2.1 and Definition 2.2. Typically, $${{\,\textrm{Ob}\,}}(C)$$ is the set of non-faulty processes, but could be extended to also include certain faulty processes, like ones that commit send omission faults only [9, 36]. The information who is obedient in a given configuration is usually not accessible to the processes, i.e., is recorded only in the corresponding global system state. We make the restriction that disobedient processes cannot recover and become obedient again, i.e., that $${{\,\textrm{Ob}\,}}(C) \supseteq {{\,\textrm{Ob}\,}}(C')$$ if $C^{\prime }$ is reachable from *C*. We extend the obedience function to the set $$\Sigma \subseteq \mathcal {C}^\omega $$ of admissible executions in a given model by setting $${{\,\textrm{Ob}\,}}:\Sigma \rightarrow 2^\Pi $$, $${{\,\textrm{Ob}\,}}(\gamma ) = \bigcap _{t\ge 0} {{\,\textrm{Ob}\,}}(C^t)$$ where $$\gamma = (C^t)_{t\ge 0}$$. Consequently, a process is obedient in an execution if it is obedient in all of its configurations. We further make the restriction that there is at least one obedient process in every execution, i.e., that $${{\,\textrm{Ob}\,}}(\gamma )\ne \emptyset $$ for all $$\gamma \in \Sigma $$.

We also assume that every process has the possibility to weakly count the steps it has taken. Formally, we assume the existence of weak clock functions $\chi _p:\mathcal {C}\rightarrow \mathbb {N}_0$ such that for every execution $\delta = (D^t)_{t\ge 0}\in \Sigma$ and every configuration $C\in \mathcal {C}$, the relation $C\sim _p D^t$ implies $t\ge \chi _p(C)$. Additionally, we assume that $\chi _p(D^t)\rightarrow \infty$ as $t\rightarrow \infty$ for every execution $\delta \in \Sigma$ and every obedient process $p\in {{\,\textrm{Ob}\,}}(\delta )$. $\chi _p$ thus ensures that a configuration $D^t$ where *p* has some specific view $V_p(D^t)=V_p(C)$ cannot occur before time $t=\chi _p(C)$ in any execution δ. Our weak clock functions hence allow us to model lockstep synchronous rounds by choosing $\chi (D^t)=t$ for any execution $\delta = (D^t)_{t\ge 0}\in \Sigma$, but are also suitable for modeling non-lockstep, even asynchronous, executions, see [32, App. A] for the implementation details.

We assume that the initial configuration $C^0$ of any execution $\gamma =(C^t)_{t\ge 0}$ contains, for every process $p \in \Pi$, a local *input value* $I_p=I_p(\gamma ) \in \mathcal {V}$ (also called *initial value*) taken from some finite set $\mathcal {V}$, collectively termed *input assignment* I(γ). Moreover, every configuration also contains a local *output value* $O_p \in \mathcal {V}\cup \{\perp \}$ (also called *decision value*). Note that $I_p$ and $O_p$ are locally accessible for *p* only, i.e., each process only knows its own initial value (and those it has heard from in the execution), and $O_p=\perp \not \in \mathcal {V}$ is used to represent the fact that *p* has not decided yet. One usually (but not necessarily, see e.g. [37]) makes the additional assumption that all input assignments with values taken from $\mathcal {V}$ are possible, as formalized in the *independent arbitrary input assignment* (IAIA) condition in Definition 6.2. A *decision algorithm* augments our full-information protocol with a collection of *decision functions* $\Delta _p:\mathcal {C}\rightarrow \mathcal {V}\cup \{\perp \}$ for $p \in \Pi$, such that $\Delta _p(C) = \Delta _p(D)$ if $C \sim _p D$, i.e., decisions depend on local information only and are deterministic.

We proceed with the specification of non-uniform and uniform consensus [28, 38] with weak or strong validity. Decision functions for terminating consensus have the additional property $$\Delta _p(C') = \Delta _p(C)$$ if $C^{\prime }$ is reachable from *C* and $$\Delta _p(C)\ne \perp $$, i.e., decisions are irrevocable. Since process *p* thus has at most one decision value in an execution, one can extend the domain of decision functions from configurations to executions by setting $$\Delta _p:\Sigma \rightarrow \mathcal {V}\cup \{\perp \}$$, $$\Delta _p(\gamma ) = \lim _{t\rightarrow \infty } \Delta _p(C^t)$$ where $$\gamma = (C^t)_{t\ge 0}$$. We say that *p* has decided value $$v\ne \perp $$ in configuration *C* (resp. execution γ) if $$O_p(C)=\Delta _p(C)=v$$ (resp. $$O_p(\gamma )=\Delta _p(\gamma )=v$$).

### Definition 2.1

*(Non-uniform and uniform terminating consensus).* A *non-uniform terminating consensus* protocol $\mathcal {A}$ is a decision algorithm that ensures the following properties in all of its admissible executions:

(T) Eventually, every obedient process must irrevocably decide. (Termination)
(A) If two obedient processes have decided, then their decision values are equal. (Agreement)
(V) If the initial values of the processes are all equal to *v*, then *v* is the only possible decision value. (Validity)

In a *strong terminating consensus* protocol $\mathcal {A}$, weak validity (V) is replaced by

(SV) The decision value must be the input value of some process. (Strong Validity)

A *uniform terminating consensus* protocol $\mathcal {A}$ must ensure (T), either (V) or (SV), and

(UA) If two processes have decided, then their decision values are equal. (Uniform Agreement)

The specification of the corresponding variants of stabilizing consensus is given in Definition 2.2. Irrevocability and the non-decided output value ⊥ are dropped here, i.e., a process *p* may change $O_p \in \mathcal {V}$ finitely many times, albeit it needs to stabilize on some common decision value $O_p = \Delta _p(\gamma )=v \in \mathcal {V}$ eventually, which does not change any more. Again, we will say that *p* has decided on value *v* in configuration *C* (resp. in execution γ) if $O_p(C)=\Delta _p(C)=v$ (resp. $O_p(\gamma )=\Delta _p(\gamma )=v$).

### Definition 2.2

*(Stabilizing consensus).* A *stabilizing consensus* protocol $\mathcal {A}$ is a decision algorithm that ensures the following properties in all of its admissible executions:

(SA) Eventually, all obedient processes must stabilize on a common decision value $v \in \mathcal {V}$. (Stabilizing Agreement)
(V) If the initial values of processes are all equal to *v*, then *v* is the only possible decision value. (Validity)

In a *strong stabilizing consensus* protocol $\mathcal {A}$, weak validity (V) is replaced by

(SV) Any decision value must be the input value of some process. (Strong Validity)

Note that, for terminating consensus, considering Uniform Agreement (UA) makes sense, since it forces a process who manages to set $O_p\ne \perp$ to assign the common decision value when doing so in that operation—even when *p* is non-obedient and thus may crash later on, for example. For stabilizing consensus, however, there is no meaningful uniform variant of Stabilizing Agreement (SA): ⊥ is not used, and it would not make sense to restrict the choices for $O_p$ for a non-obedient process at any finite time.

For both terminating and stabilizing consensus, (A) as well as (SA) and the fact that every execution has at least one obedient process allow us to define a *global decision function* $\Delta : \Sigma \rightarrow \mathcal {V}$, by setting $\Delta (\gamma ) = \Delta _p(\gamma )$ for an arbitrary process *p* that is obedient in execution γ, i.e., $p\in {{\,\textrm{Ob}\,}}(\gamma )$.

## Basics point-set-topology

We first recall briefly the basic topological notions that are needed for our exposition; an in-depth treatment can be found in [39], for example.

A *topology* on a set *X* is a family $\mathcal {T}$ of subsets of *X* such that $\emptyset \in \mathcal {T}$, $X \in \mathcal {T}$, and $\mathcal {T}$ contains all arbitrary unions as well as all finite intersections of its members. We call *X* endowed with $\mathcal {T}$, often written as $(X, \mathcal {T})$, a *topological space* and the members of $\mathcal {T}$ *open sets*. For example, if *X* is endowed with the *discrete topology*, every subset A⊆X is open. The complement of an open set is called *closed* and sets that are both open and closed, such as ∅ and *X* itself, are called *clopen*.

Given a space $(X, \mathcal {T})$, Y⊆X is called a subspace of *X* if *Y* is equipped with the subspace topology $\{ Y \cap U \mid U \in \mathcal {T}\}$. A space *X* is called *compact* if every family of open sets that covers *X* contains a finite sub-family that covers *X*. A point x∈X in a topological space *X* is an *isolated point* if the singleton set $\{x\}$ is open.

Given A⊆X, the *closure* $\overline{A}$ of *A* in *X* is the intersection of all closed sets containing *A*. Note that if Y⊆X is a subspace of *X* and a set A⊆Y, the closure $\widehat{A}$ of *A* in *Y* is equal to $\widehat{A}=Y \cap \overline{A}$. The *interior* ${{\,\textrm{Int}\,}}(A)$ of *A* is the union of all open sets contained in *A*. The *boundary* of *A* is defined as ${{\,\mathrm{\partial }\,}}A=\overline{A}-{{\,\textrm{Int}\,}}(A)$. A set *A* is called *dense* in *X*, if $\overline{A}=X$. A set *A* is *nowhere dense* in *X*, if ${{\,\textrm{Int}\,}}(\overline{A})=\emptyset$. Note that (the closure of) a nowhere dense set *A* does not contain any non-empty open set, and that the complement of $\overline{A}$ is dense in *X*. The closure of a nowhere dense set, every subset of a nowhere dense set, and a finite union of nowhere dense sets can be shown to be nowhere dense. Nowhere dense sets can also be characterized by the following property:

### Lemma 3.1

A set *A* of a topological space *X* is nowhere dense in *X* if and only if every open set *U* contains a non-empty open subset V⊆U satisfying $V\cap A = \emptyset$.

For a space *X*, if A⊆X, we call *x* a *limit point* of *A*, if any open set *U* containing *x* intersects *A* in a point different from *x*; this is equivalent to requiring that *x* belongs to the closure of $$A \setminus \{ x \}$$. The set of all limit points of *A* is denoted $A^{\prime }$, which is also called the *derived set* of *A*. It can be shown that $$\overline{A}=A \cup A' = A \cup {{\,\mathrm{\partial }\,}}A $$. Note that $${{\,\mathrm{\partial }\,}}A$$ may also contain certain points in *A*, namely, ones that are neither interior nor limit points (in particular, isolated points).

A topological space *X* is *disconnected*, if it contains a nontrivial clopen set, which means that it it can be partitioned into two disjoint open sets. It is *connected* if it is not disconnected. The *connected components* of a space *X* are connected disjoint subspaces of *X* whose union is *X*, such that each nonempty connected subspace of *X* intersects only one of them. Since the closure of a connected subspace of *X* is connected, every connected component of *X* is closed.

A function *f* from space *X* to space *Y* is *continuous* if the pre-image of every open set in *Y* is open in *X*. We say that *f* is *continuous at the point* x∈X if for every open set V⊆Y containing *f*(*x*) there is an open set U⊆X containing *x* such that f(U)⊆V. A *point of discontinuity* of *f* is a point x∈X where *f* is not continuous, i.e., where there is some open set *V* with f(x)∈V but $f(U) \not \subseteq V$ for every open set *U* with x∈U.

If *X* is a nonempty set, then we call any function $d:X\times X\rightarrow \mathbb {R}_+$ a *distance function* on *X*. Many topological spaces are defined by *metrics*, which are distance functions that are *definite* (d(x,y)=0 iff x=y for all x,y∈X), *symmetric* (d(x,y)=d(y,x) for all x,y∈X) and satisfy the *triangle inequality* (d(x,z)≤d(x,y)+d(y,z) for all x,y,z∈X). For a distance function to define some (potentially non-metrizable) topology though, no additional assumptions are necessary: One can define $\mathcal {T}_d \subseteq 2^X$ by setting $U\in \mathcal {T}$ if and only if for all x∈U there exists some ε>0 such that $B_\varepsilon (x) = \{ y \in X \mid d(x,y) < \varepsilon \} \subseteq U$.

The *product topology* on a *product space* $\Pi _{\iota \in I} X_\iota$ of topological spaces is defined as the coarsest topology such that all projections $\pi _i:\Pi _{\iota \in I}X_\iota \rightarrow X_i$ are continuous. A topology $(X,\mathcal {T})$ is *coarser* than a topology $(X,\mathcal {T}')$ if every open set in $\mathcal {T}$ is also open in $\mathcal {T}'$. In that case, the topology $(X,\mathcal {T}')$ is called *finer* than $(X,\mathcal {T})$, as it may contain additional open sets that are not open in the latter. It was shown in [32] that the product topology on the space $\mathcal {C}^\omega$, i.e., infinite sequences of configurations making up executions, is induced by the following distance function (where the particular instances of *d* will be defined in Eq. (2), Eq. (4) and Eq. (5) in Section 4):

### Lemma 3.2

Let *d* be a distance function on *X* that only takes the values 0 or 1. Then the product topology of $X^\omega$, where every copy of *X* is endowed with the topology induced by *d*, is induced by the distance function $d_d$ defined as

1 $$\begin{aligned} d_d: X^\omega \times X^\omega \rightarrow \mathbb {R}\quad ,\quad d_d(\gamma ,\delta )= 2^{-\inf \{t\ge 0\mid d(C^t,D^t) > 0\}} \end{aligned}$$

where $$\gamma = (C^t)_{t\ge 0}$$ and $$\delta = (D^t)_{t\ge 0}$$.

Distance functions are generalized to sets A,B⊆X by defining $d(A,B)=\inf \{d(\alpha ,\beta )\mid \alpha \in A,\,\beta \in B\}$.

Equipped with these prerequisites, we can re-state some key results of Levine’s study [34] of semi-open sets and semi-continuous functions, which will turn out to be a perfect fit for characterizing stabilizing consensus.

### Definition 3.3

**Fig. 1:**
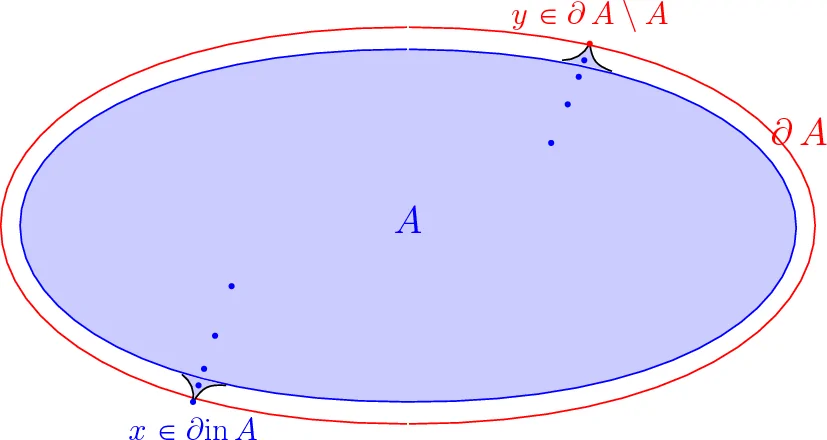
Illustration of a semi-open set *A* with boundary ${{\,\mathrm{\partial }\,}}A$. The limit point $x \in {{\,\mathrm{\partial }\,}}A$ is included in *A*, so $x\in {{\,\mathrm{\partial in}\,}}A$, whereas the limit point $y\in {{\,\mathrm{\partial }\,}}A$ is not included in *A*. In general, *A* may include any number of such limit points, including all or none.

*(Semi-open sets* [34, Def. 1], *see Fig.* 1*).* A set A in a topological space *X* will be termed semi-open (written s.o.) if and only if there exists an open set *O* such that $O \subseteq A \subseteq \overline{O}$.

### Theorem 3.4

( [34, Thm. 2]). Let $\{A_\alpha \}_{\alpha \in \Delta }$ be a collection of s.o. sets in a topological space *X*. Then $\bigcup _{\alpha _\in \Delta } A_\alpha$ is s.o.

### Theorem 3.5

( [34, Thm. 6]). Let $A \subseteq Y \subseteq X$ where *X* is a topological space and *Y* is a subspace. Let *A* be s.o. in *X*. Then *A* is s.o. in *Y*.

### Lemma 3.6

( [34, Lem. 1]). Let *O* be open in *X*. Then ${{\,\mathrm{\partial }\,}}O = \overline{O}-O$ is nowhere dense in *X*.

We introduce the abbreviation ${{\,\mathrm{\partial in}\,}}A$ for boundary points included in *A*, which allows us to write $S=O \cup {{\,\mathrm{\partial in}\,}}S$ for the open set $O={{\,\textrm{Int}\,}}{S}$ guaranteeing $O \subseteq S \subseteq \overline{O}$ in the case of a semi-open set *S*:

### Definition 3.7

*(Included boundary points).* The set of included boundary points of A⊆X of a topological space *X* is ${{\,\mathrm{\partial in}\,}}A = A \cap {{\,\mathrm{\partial }\,}}A$.

### Theorem 3.8

([34, Thm. 7]). Let *A* be s.o. in *X*. Then A=O∪B where (1) *O* is open in *X*, (2) $O \cap B = \emptyset$ and (3) *B* is nowhere dense.

In general, the converse of Theorem 3.8 is false, i.e., not every nowhere dense set is an acceptable $B={{\,\mathrm{\partial in}\,}}A$ for a semi-open set *A*. The following choice is feasible, though:

### Theorem 3.9

([34, Thm. 8]). Let *X* be a topological space and A=O∪B where (1) $$O\ne \emptyset $$ is open in *X*, (2) *A* is connected and (3) $B^{\prime }=\emptyset$ where $B^{\prime }$ is the derived set [i.e., the set of all limit points] of *B*. Then *A* is s.o.

The following definition introduces semi-continuity as a weaker form of continuity, in the sense that a continuous map is also semi-continuous but not necessarily vice versa:

### Definition 3.10

*(Semi-continuity* [34, Def. 4]*)*. Let $f: X \rightarrow X^*$ be single-valued (continuity not assumed) where *X* and $X^*$ are topological spaces. Then $f: X \rightarrow X^*$ is termed semi-continuous (written s.c.) if and only if, for $O^*$ open in $X^*$, then $f^{-1}(O^*)$ is semi-open in *X*.

The following theorem is crucial for characterizing semi-continuous functions:

### Theorem 3.11

([34, Thm. 12]). Let $f: X \rightarrow X^*$ be a single-valued function, *X* and $X^*$ being topological spaces. Then $f: X \rightarrow X^*$ is s.c. if and only if for [some open set $O^*$ with] $f(p)\in O^*$, there exists an *A* that is semi-open in *X* such that p∈A and $f(A) \subseteq O^*$.

## Topological characterization of terminating consensus

In this section, we endow the set of admissible executions Σ introduced in Section 2 with the uniform and non-uniform topologies defined in [32]: Indeed, in order to be able to explain the relation between terminating and stabilizing consensus, we will re-state a few core results of this topological characterization of terminating consensus.

### *p*-view topology

Let the *p*-*view distance function* $d_p$ on the set $\mathcal {C}$ of configurations for every process $p\in \Pi$ be defined by

2 $$\begin{aligned} d_p(C,D) = {\left\{ \begin{array}{ll} 0 &  \text {if } C \sim _p D \text { and } p\in {{\,\textrm{Ob}\,}}(C)\cap {{\,\textrm{Ob}\,}}(D),\\ 1 &  \text {otherwise}. \end{array}\right. } \end{aligned}$$

Extending this distance function from configurations to executions according to Lemma 3.2, we define the *p*-*view distance function* by

3 $$\begin{aligned} d_p:\Sigma \times \Sigma \rightarrow \mathbb {R}_+,\quad d_p(\gamma ,\delta ) = 2^{-\inf \{t\ge 0\mid d_p(C^t,D^t) > 0\}}, \end{aligned}$$

where $$\gamma = (C^t)_{t\ge 0}$$ and $$\delta = (D^t)_{t\ge 0}$$.

Lemma 4.1 reveals that Eq. (3) defines a pseudometric, which differs from a metric by lacking definiteness: There may be executions $\gamma \ne \delta$ with $d_p(\gamma ,\delta ) =0$.

#### Lemma 4.1

(Pseudometric $d_p$ [32, Lem. 4.3]). The *p*-view distance function $d_p$ is a pseudometric, i.e., it satisfies:

$$\begin{aligned} d_p(\gamma ,\gamma )&=0 \\ d_p(\gamma ,\delta )&= d_p(\delta ,\gamma ) \qquad \text{(symmetry) }\\ d_p(\beta ,\delta )&\le d_p(\beta ,\gamma ) + d_p(\gamma ,\delta ) \qquad \text{(triangle } \text{ inequality) } \end{aligned}$$

#### Uniform topology

The *uniform minimum topology* (abbreviated *uniform topology*) on the set Σ of executions is induced by the distance function

4 $$\begin{aligned} d_{\textrm{u}}(\gamma , \delta ) = \min _{p\in \Pi } d_p(\gamma ,\delta ). \end{aligned}$$

Note that $d_{\textrm{u}}$ does not necessarily satisfy the triangle inequality (nor definiteness): There may be executions with $d_{p}(\beta ,\gamma )=0$ and $d_{q}(\gamma ,\delta )=0$ but $d_{r}(\beta ,\delta )>0$ for all $r\in \Pi$. Hence, the topology on Σ induced by $d_{\textrm{u}}$ lacks some of the convenient (separation) properties of metric spaces.

The next lemma shows that the decision function of a protocol that solves uniform consensus is always continuous with respect to the uniform topology. Informally, since $\mathcal {V}$ is equipped with the discrete topology, this continuity implies that the decision value $\Delta (\gamma )$ and $\Delta (\delta )$ of two executions γ, δ that are sufficiently close to each other (i.e., $d_u(\gamma ,\delta )$ is sufficiently small) satisfy $\Delta (\gamma )=\Delta (\delta )$.

##### Lemma 4.2

([32, Lem. 4.4]). Let $\Delta :\Sigma \rightarrow \mathcal {V}$ be the decision function of a uniform consensus protocol. Then, Δ is continuous with respect to the uniform distance function $d_{\textrm{u}}$.

##### Definition 4.3

*(v*-*valent execution).* We call an execution $\gamma _v \in \Sigma$, for $v\in \mathcal {V}$, *v*-valent, if it starts from an initial configuration where all processes $p\in \Pi$ have the same initial value $I_p(\gamma _v)=v$.

##### Theorem 4.4

(Characterization of uniform consensus [32, Thm. 5.2]). Uniform consensus is solvable if and only if there exists a partition of the set Σ of admissible executions into sets $\Sigma _v$, $v\in \mathcal {V}$, such that the following holds:

1. Every $\Sigma _v$ is a clopen set in Σ with respect to the uniform topology induced by $d_{\textrm{u}}$.
2. If execution $\gamma \in \Sigma$ is *v*-valent, then $\gamma \in \Sigma _v$.

#### Non-uniform topology

Whereas the *p*-view distance function given by Eq. (3) is also meaningful for non-uniform consensus, this is not the case for the uniform distance function as defined in Eq. (4). The appropriate *non-uniform minimum topology* (abbreviated *non-uniform topology*) on the set Σ of executions is induced by the distance function

5 $$\begin{aligned} d_{\textrm{nu}}(\gamma , \delta ) = {\left\{ \begin{array}{ll} \min _{p\in {{\,\textrm{Ob}\,}}(\gamma )\cap {{\,\textrm{Ob}\,}}(\delta )} d_p(\gamma ,\delta )\\ \qquad \qquad \text {if } {{\,\textrm{Ob}\,}}(\gamma )\cap {{\,\textrm{Ob}\,}}(\delta ) \ne \emptyset ,\\ 1\\ \qquad \qquad \text {if } {{\,\textrm{Ob}\,}}(\gamma )\cap {{\,\textrm{Ob}\,}}(\delta ) = \emptyset . \end{array}\right. } \end{aligned}$$

Like for $d_{\textrm{u}}$, neither definiteness nor the triangle inequality need to be satisfied by $d_{\textrm{nu}}$. The resulting non-uniform topology is finer than the uniform topology, however, since the minimum is taken over the smaller set ${{\,\textrm{Ob}\,}}(\gamma )\cap {{\,\textrm{Ob}\,}}(\delta )\subseteq \Pi$: Indeed, less executions are within the open ball $B_\varepsilon ^{\textrm{nu}}(\gamma )$ compared to $B_\varepsilon ^{\textrm{u}}(\gamma )$, i.e., $B_\varepsilon ^{\textrm{nu}}(\gamma ) \subseteq B_\varepsilon ^{\textrm{u}}(\gamma )$. Conversely, some $\delta \in B_\varepsilon ^{\textrm{u}}(\gamma )$ need not satisfy $\delta \in B_\varepsilon ^{\textrm{nu}}(\gamma )$, which implies $d_{\textrm{u}}(\gamma ,\delta ) \le d_{\textrm{nu}}(\gamma ,\delta )$. It follows that every decision function that is continuous with respect to the uniform topology is also continuous with respect to the non-uniform topology.

##### Lemma 4.5

([32, Lem. 4.5]). Let $\Delta :\Sigma \rightarrow \mathcal {V}$ be the decision function of a non-uniform consensus protocol. Then, Δ is continuous with respect to the non-uniform distance function $d_{\textrm{nu}}$.

##### Theorem 4.6

(Characterization of non-uniform consensus [32, Thm. 5.3]). Non-uniform consensus is solvable if and only if there exists a partition of the set Σ of admissible executions into sets $\Sigma _v$, $v\in \mathcal {V}$, such that the following holds:

1. Every $\Sigma _v$ is a clopen set in Σ with respect to the non-uniform topology induced by $d_{\textrm{nu}}$.
2. If execution $\gamma \in \Sigma$ is *v*-valent, then $\gamma \in \Sigma _v$.

#### The importance of limit points

Additional results established in [32] reveal that certain limit points play a crucial role for consensus solvability. Indeed, since consensus is a continuous function (Lemma 4.2 and Lemma 4.5), it is not too surprising that consensus is impossible if and only if limit points “between” different decision sets $\Sigma _v$ and $\Sigma _w$, i.e., joint limit points, are admissible.

Applying the following well-known topological Lemma 4.7 to the findings of Theorem 4.4 (resp. Theorem 4.6) yields the limit-based characterization of consensus solvability given in Corollary 4.8.

##### Lemma 4.7

(Separation Lemma [39, Lemma 23.12]). If *Y* is a subspace of *X*, a separation of *Y* is a pair of disjoint nonempty sets *A* and *B* whose union is *Y*, neither of which contains a limit point of the other. The space *Y* is connected if and only if there exists no separation of *Y*. Moreover, *A* and *B* of a separation of *Y* are clopen in *Y*.

##### Theorem 4.8

(Separation-based consensus characterization [32, Thm. 6.4]). Uniform (resp. non-uniform consensus) is solvable in a model if and only if there exists a partition of the set of admissible executions Σ into decision sets $\Sigma _v,v\in \mathcal {V}$, such that the following holds:

1. No $\Sigma _v$ contains a limit point of any other $\Sigma _w$ w.r.t. the uniform (resp. non-uniform) topology in $\mathcal {C}^{\omega }$.
2. Every *v*-valent admissible execution $\gamma _v$ satisfies $\gamma _v\in \Sigma _v$.

If consensus is not solvable, then $d_{\textrm{u}}(\Sigma _v,\Sigma _w)=0$ (resp. $d_{\textrm{nu}}(\Sigma _v,\Sigma _w)=0$) for some w≠v.

Corollary 4.8 can also be expressed via the exclusion of fair/unfair executions as defined in [40]:

##### Definition 4.9

*(Fair and unfair executions* [32, Def. 6.6]*)*. Consider two executions $\rho , \rho ' \in \mathcal {C}^{\omega }$ of some consensus algorithm with decision sets $\Sigma _v$, $v\in \mathcal {V}$, in any appropriate topology [i.e., $d_{\textrm{u}}$, $d_{\textrm{nu}}$ or $d_p$]:

- ρ is called *fair*, if for some $v,w\ne v \in \mathcal {V}$ there are convergent sequences $(\alpha _k) \in \Sigma _v$ and $(\beta _k) \in \Sigma _w$ with $\alpha _k\rightarrow \rho$ and $\beta _k\rightarrow \rho$.
- ρ, $\rho^{\prime }$ are called a pair of *unfair* executions, if for some $$v, w \in \mathcal {V}$$, w≠v, there are convergent sequences $$(\alpha _k) \in \Sigma _v$$ with $$\alpha _k\rightarrow \rho $$ and $$(\beta _k) \in \Sigma _w$$ with $$\beta _k\rightarrow \rho '$$ and ρ and $\rho^{\prime }$ have distance 0.

##### Corollary 4.10

(Fair/unfair consensus characterization [32, Cor. 6.7]). Condition (1) in Corollary 4.8 is equivalent to requiring that the decision sets $\Sigma _v$, $\Sigma _w$ for w≠v neither contain any fair execution nor any pair $\rho ,\rho '$ of unfair executions.

An illustration of our limit-based characterizations is provided by Figure 3.

**Fig. 2:**
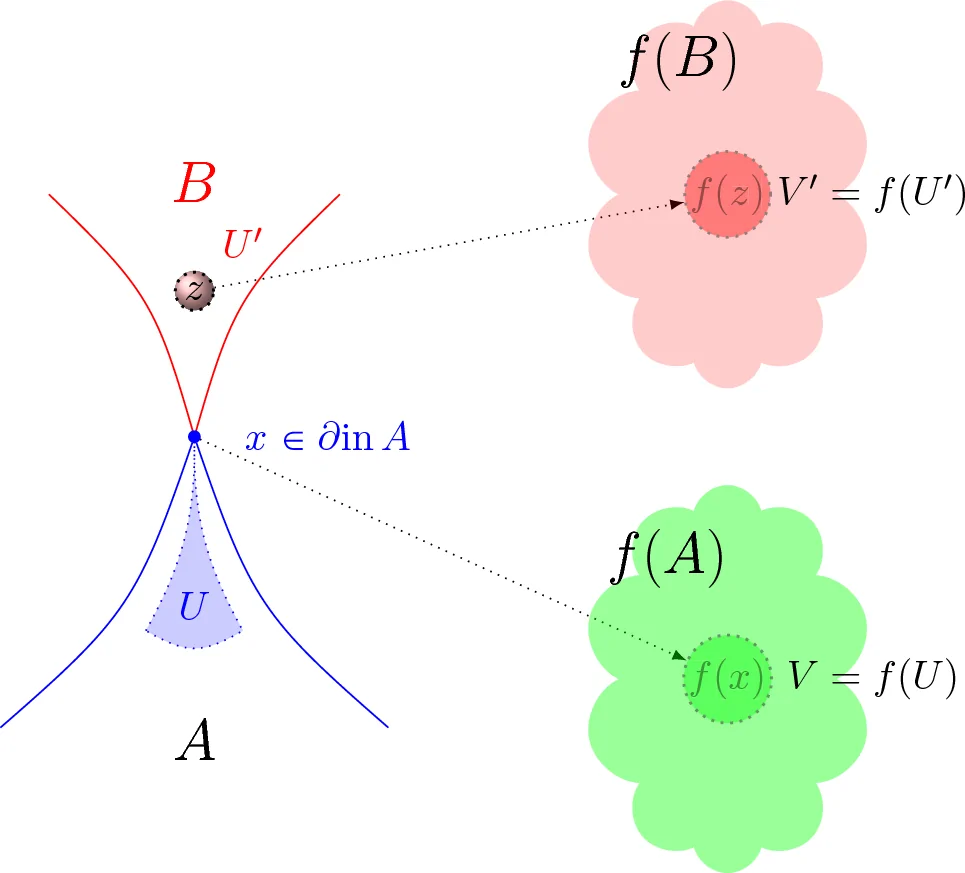
Illustration of a semi-continous function $$f:X \rightarrow X^*$$. On the left, there is a semi-open set $$A \cup B \subseteq X$$ consisting of two sets *A* and *B* that share a common limit point $$x \in {{\,\mathrm{\partial }\,}}A \cap {{\,\mathrm{\partial }\,}}B$$, which belongs to $${{\,\mathrm{\partial in}\,}}A$$. In the upper part, a point *z* is mapped to f(z)∈f(B) such that pre-image of the open set $V^{\prime }\subseteq f\left(B\right)$ is an open set $U^{\prime }\subseteq B$ that contains *x*; in fact, *f* is even *continuous* at *z*. By contrast, the limit point *x* is mapped to f(x)∈f(A) lying in some open set V⊆f(A). Since *f* is semi-continous, the pre-image *U* only needs to be semi-open. Note that the image of *no* open ball centered at *x* would lie solely in *f*(*A*) here.

**Fig. 3:**
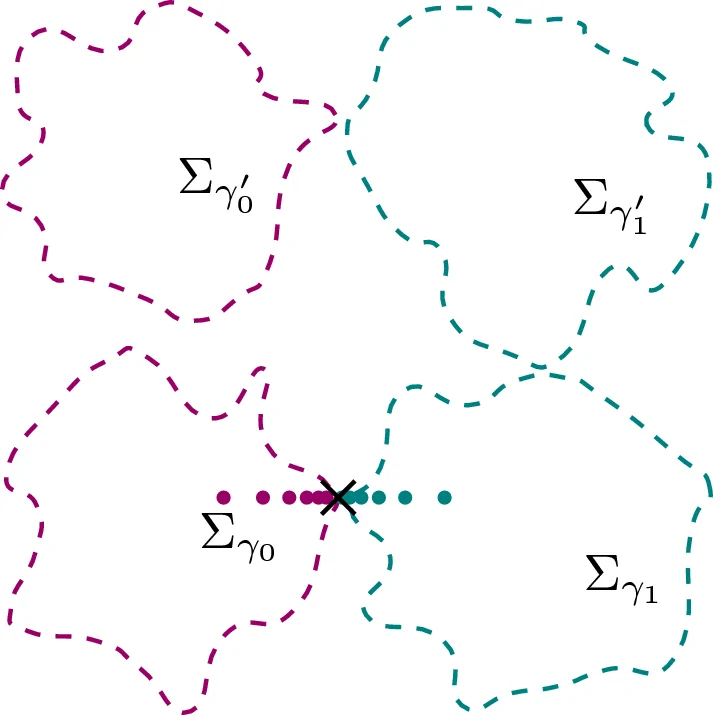
Illustration of two connected components of the decision sets $\Sigma _0=\Sigma _{\gamma _0}\cup \Sigma _{\gamma _0'}$ and $\Sigma _1=\Sigma _{\gamma _1}\cup \Sigma _{\gamma _1'}$, taken from [32, Fig. 4]. Common limit points of two connected components from different decision sets (like $\Sigma _{\gamma _0}$ and $\Sigma _{\gamma _1}$, marked by ×) must not be admissible by Corollary 4.8 to guarantee terminating consensus solvability.

## Topological characterization of stabilizing consensus

In this section, we will use the non-uniform topology introduced in Section 4 in conjunction with the topological results on semi-open sets and semi-continuous functions developed by Levine in [34] (which we re-stated in Section 3) for characterizing the solvability of stabilizing consensus. Note that, since there is no meaningful uniform variant of Stabilizing Agreement (SA) in Definition 2.2, as already mentioned in Section 2, the uniform topology would not be useful here.

We already noted at the beginning of Section 4 that the continuity of the decision function of terminating consensus makes it mandatory that there is no admissible limit point “between” two different decision sets, recall the illustration in Fig. 3. After all, a continous function cannot map a connected space to a disconnected space (cp. Fig. 2). For example, since all executions are admissible in the lossy-link model, resulting in a connected space $\Sigma =\mathcal {C}^\omega$, it is hence impossible to solve terminating consensus.

Since stabilizing consensus *can* be solved in the lossy-link model (see Section 7.2.1), its decision function cannot be continuous. Rather, it must be able to map a connected space to a disconnected one. Lemma 5.3 below will reveal that the decision function of a *proper* stabilizing consensus protocol must be *semi-continuous*, in the sense of Definition 3.10, and that the induced decision sets must be *semi-open*, in the sense of Definition 3.3.

Clearly, like in the case of terminating consensus, the decision function of any (correct) stabilizing consensus protocol induces a unique partition of the set of admissible executions into disjoint decision sets $\Sigma =\bigcup _{v\in \mathcal {V}} \Sigma _v$. *Proper* decision functions will ensure that no decision set $\Sigma _v$ contains an execution that is *not* one of its own limit points (but rather a limit point of some other decision set $\Sigma _w$):

### Definition 5.1

*(Proper stabilizing consensus).* A decision function for solving stabilizing consensus is called *proper*, iff none of its induced decision sets $\Sigma _v$, $v\in \mathcal {V}$, contains any execution $\gamma \not \in \overline{{{\,\textrm{Int}\,}}(\Sigma _v)}$, unless γ is an isolated point in Σ (where $\{\gamma \}$ is open in Σ).

Note that an isolated point in Σ is also an isolated point in the subspace $\Sigma _v$, but not vice versa. Indeed, properness prohibits the inclusion of an execution β in $\Sigma _v$, which is a limit of some sequence $\beta _1,\beta _2, \dots \in \Sigma _w$, but not a limit of any sequence $\alpha _1,\alpha _2, \dots \in \Sigma _v$. If included in $\Sigma _v$ nevertheless, this β would constitute an isolated point in the subspace $\Sigma _v$, but *not* in Σ, so Definition 5.1 prohibits this inclusion.

Whereas Definition 5.1 restricts the class of stabilizing consensus protocols covered by our topological characterization, we will prove later (Lemma 5.8) that it is easy to turn a decision function $\Delta^{\prime }$ of a non-proper stabilizing consensus protocol into a proper decision function Δ.

We first instantiate Theorem 3.11 in our special context, which exploits the fact that every singleton set $\{v\}$ for $v\in \mathcal {V}$ is open in the discrete topology.

### Corollary 5.2

(Characterization of semi-continuous functions). Let $\Delta : \Sigma \rightarrow \mathcal {V}$ (where Σ is equipped with the non-uniform topology, and $\mathcal {V}$ is equipped with the discrete topology) be a single-valued function. Then $\Delta : \Sigma \rightarrow \mathcal {V}$ is semi-continuous if and only if for every execution $\gamma \in \Sigma$ with $\Delta (\gamma )=v\in \mathcal {V}$, there exists a semi-open set *A* in Σ such that $\gamma \in A$ and Δ(A)=v.

### Lemma 5.3

Let $\Delta :\Sigma \rightarrow \mathcal {V}$ be the decision function of a proper stabilizing consensus protocol. Then, Δ is semi-continuous with respect to the non-uniform distance function $d_{\textrm{nu}}$.

### Proof

We use the only-if direction (⇐) of Corollary 5.2. Let $\Sigma _v=\Delta ^{-1}(v)$, and distinguish 3 cases for $\gamma \in \Sigma _v$ when determining the required semi-open set $A \subseteq \Sigma _v$ containing γ:

(1) If $\gamma \in {{\,\textrm{Int}\,}}(\Sigma _v)$, there must be some k≥0 such that the open ball $B_{2^{-k}}(\gamma )$ with radius $2^{-k}$ (for distance function $d_{\textrm{nu}}$) satisfies $B_{2^{-k}}(\gamma ) \subseteq \Sigma _v$. We take $A=B_{2^{-k}}(\gamma )$ for Corollary 5.2 in this case, which is obviously open and hence semi-open.

(2) If γ is an isolated point in $\Sigma _v$, the singleton set $\{\gamma \}$ is open in $\Sigma _v$, so we can safely take $A=\{\gamma \}$, which is again open and hence semi-open.

(3) If $\gamma \in {{\,\mathrm{\partial in}\,}}\Sigma _v$ is an (included) boundary point and hence a limit point of $\Sigma _v$ (as Δ is a proper stabilizing consensus decision function), every open ball $B_{2^{-k}}(\gamma )$, k≥0, also intersects $\Sigma _v$ in a point $\beta _k$ different from γ. Choosing $\beta _k \in {{\,\textrm{Int}\,}}(\Sigma _v)$, there is some $\ell _k\ge 0$ such that the open ball $B_{2^{-\ell _k}}(\beta _k) \subseteq {{\,\textrm{Int}\,}}(\Sigma _v)$. We thus take $A=\{\gamma \}\cup \bigcup _{k\ge 0}B_{2^{-\ell _k}}(\beta _k)$, which is semi-open as an arbitrary union of (semi-)open sets with limit point γ, recall Theorem 3.4.

Applying the only-if direction of Corollary 5.2 proves the asserted semi-continuity of Δ. □

By applying Lemma 3.6 to the result of Lemma 5.3 while recalling Definition 5.1, we immediately observe that the boundaries of all decision sets are nowhere dense:

### Corollary 5.4

(Nowhere dense boundaries). Let $\Delta : \Sigma \rightarrow \mathcal {V}$ be a proper stabilizing consensus decision function, and $\Sigma _v$, $v\in \mathcal {V}$ be the corresponding decision sets. Then, the boundary ${{\,\mathrm{\partial }\,}}\Sigma _v$, and hence the included boundary ${{\,\mathrm{\partial in}\,}}\Sigma _v$, of every decision set is nowhere dense.

Since the union of the boundaries ${{\,\mathrm{\partial }\,}}\Sigma _v$ of all decision sets contains the points of discontinuity of Δ, Lemma 5.4 is consistent with the result of the following Lemma 5.5, which is a specialization of [34, Thm. 13]:

### Theorem 5.5

(Nowhere dense discontinuities). Let $\Delta : \Sigma \rightarrow \mathcal {V}$ be a proper stabilizing consensus decision function. Then, the set *P* of points of discontinuity of Δ in Σ is nowhere dense.

### Proof

Since $\mathcal {V}$ is equipped with the discrete topology, it has a finite open basis since the singleton sets $\{v\}$, $v\in \mathcal {V}$, are open. For every point of discontinuity $\gamma \in P$, there exists an open set $V_\gamma =\{v\}$ for some $v\in \mathcal {V}$, such that for some open set $W \subseteq \Sigma$ it holds that $\gamma \in W$ but $\Delta (W) \not \subseteq V_\gamma$. By Corollary 5.2, there exists a semi-open set $A_\gamma$ with $\gamma \in A_\gamma$ and $\Delta (A_\gamma ) \subseteq \{v\}=V_\gamma$. By definition, $A_\gamma = O_\gamma \cup B_\gamma$ with $B_\gamma \subseteq {{\,\mathrm{\partial }\,}}O_\gamma = \overline{O_\gamma }-O_\gamma$, so $\gamma \not \in O_\gamma$ and hence $\gamma \in B_\gamma$. Since ${{\,\mathrm{\partial }\,}}O_\gamma$ is nowhere dense by Lemma 3.6, the subset $B_\gamma$ is also nowhere dense. As there are only finitely many different sets $V_\gamma$, it follows that $\bigcup _{\gamma \in P}B_\gamma$ is a finite union of nowhere dense sets, which is also nowhere dense. Since $P \subseteq \bigcup _{\gamma \in P}B_\gamma$, this also holds for *P*. □

To simplify the proof of the main result of this section, namely our topological characterization of stabilizing consensus solvability given in Theorem 5.7 below, we provide a technical lemma first: Assuming that some obedient process *p* executes its *i*-th computing step at some time $t_i\ge 0$ in an a priori unknown admissible execution $\gamma = (C^t)_{t\ge 0} \in \Sigma$ of a proper stabilizing consensus protocol, Lemma 5.6 tells something about the set of executions

6 $$\begin{aligned} D_p(\gamma ,{t_i})&= \bigl \{\delta =(D^t)_{t\ge 0} \in \Sigma \mid (p \in {{\,\textrm{Ob}\,}}(\delta ))\nonumber \\&\qquad \qquad \qquad \qquad \wedge \exists s: C^{t_i} \sim _p D^s\bigr \} \nonumber \\&= \bigl \{\delta =(D^t)_{t\ge 0} \in \Sigma \mid (p \in {{\,\textrm{Ob}\,}}(\delta )) \nonumber \\&\qquad \qquad \qquad \qquad \wedge \exists s: V_p(C^{t_i})= V_p(D^s)\bigr \}. \end{aligned}$$

that process *p* cannot distinguish from the execution γ based on its local view:

### Lemma 5.6

The set of admissible executions $D_p(\gamma ,{t_i})$ defined in Eq. (6) satisfies the following properties:

(i) For every i≥0, $D_p(\gamma ,{t_i})$ can be locally computed by process *p*.
(ii) For every i≥0, $\gamma \in D_p(\gamma ,{t_i})$.
(iii) If γ is an interior point of some $\Sigma _v$, $v\in \mathcal {V}$, or a boundary point $\gamma \in {{\,\mathrm{\partial in}\,}}\Sigma _v$ satisfying $\gamma \not \in {{\,\mathrm{\partial }\,}}\Sigma _w$ for w≠v, then there is some global time L≥0 (resp. some index $\ell \ge 0$), such that, for every $i\ge \ell$,
  7 $$\begin{aligned} D_p(\gamma ,{t_i}) \subseteq B_{2^{-\ell }}^p(\gamma ) \subseteq B_{2^{-\ell }}(\gamma ) \subseteq \Sigma _v. \end{aligned}$$
  $D_p(\gamma ,{t_i})$ is hence open both in the *p*-view topology and in the non-uniform topology for every time $t_i \ge L$ (resp. for every index $i\ge \ell$).

### Proof

As for (i), *p* can compute the set $D_p(\gamma ,{t_i})$ at any time ${t_i}$, since it depends only on the current view $V_p(C^{t_i})$ and the sets $\Sigma _v$, $v\in \mathcal {V}$, which we assume to be a priori known to *p*.

Property (ii) is trivially satisfied, since $\delta =\gamma$ is always a feasible choice of an indistinguishable execution in Eq. (6).

To finally prove (iii), we assume that $\gamma \in {{\,\textrm{Int}\,}}(\Sigma _v)$ is an interior point, or $\gamma \in {{\,\mathrm{\partial in}\,}}\Sigma _v$ with $\gamma \not \in {{\,\mathrm{\partial }\,}}\Sigma _w$ for w≠v. In both cases, there exists some $\ell \ge 0$ such that the open ball $B_{2^{-\ell }}(\gamma )=\left\{ \delta \in \Sigma \mid d_{\textrm{nu}}(\gamma ,\delta ) < 2^{-\ell } \right\} \subseteq \Sigma _v$. By definition of $d_{\textrm{nu}}$, it follows that $d_{\textrm{nu}}(\gamma ,\delta ) \le d_p(\gamma ,\delta )$ and hence $B_{2^{-\ell }}^p(\gamma )=\bigl \{ \delta \in \Sigma \mid (p \in {{\,\textrm{Ob}\,}}(\delta )) \wedge d_p(\gamma ,\delta ) < 2^{-\ell } \bigr \} \subseteq B_{2^{-\ell }}(\gamma ) \subseteq \Sigma _v$.

Recalling $\gamma = (C^t)_{t\ge 0}$, let *L* be the smallest integer such that $2^{-\chi _p(C^t)} \le 2^{-\ell }$ for all t≥L. Such an *L* exists, since $\chi _p(C^t)\rightarrow \infty$ as $t\rightarrow \infty$ by the properties of our weak clock function χ defined in Section 2. Then, for every ${t_i}\ge L$, we find

$$\begin{aligned} D_p(\gamma ,{t_i})&= \left\{ \delta \in \Sigma \mid (p \in {{\,\textrm{Ob}\,}}(\delta )) \wedge \exists s:C^{t_i} \sim _p D^s \right\} \\&\subseteq \left\{ \delta \in \Sigma \mid (p \in {{\,\textrm{Ob}\,}}(\delta )) \wedge d_p(\gamma ,\delta ) < 2^{-\chi _p(C^{t_i})} \right\} \\&\subseteq B_{2^{-\ell }}^p(\gamma ) \subseteq B_{2^{-\ell }}(\gamma ) \subseteq \Sigma _v \end{aligned}$$

Note that, since the sequence $(t_i)_{i\ge 0}$ is by definition monotonically increasing, the existence of some $t_i \ge L$ translates into the relation $i \ge \ell$ for the appropriate index. Since Eq. (7) reveals that $D_p(\gamma ,{t_i})$ is contained in an open set in $\Sigma _v$, hence open, this completes our proof. □

**Figure:**
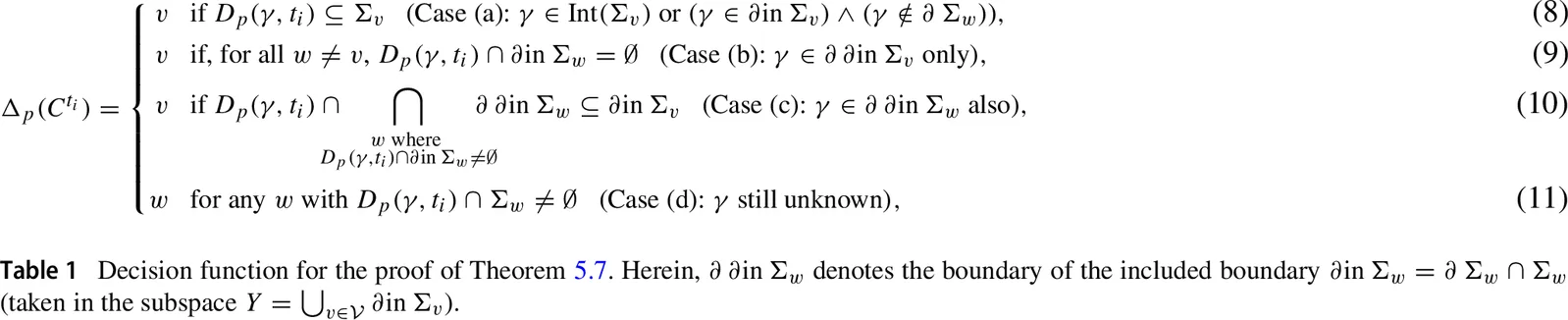

The following Theorem 5.7 provides the topological characterization of stabilizing consensus solvability in the non-uniform topology:

### Theorem 5.7

(Characterization of stabilizing consensus). Stabilizing consensus with weak validity is solvable with a proper decision function if and only if the set Σ of admissible executions can be split into disjoint decision sets $\Sigma _v$, $v\in \mathcal {V}$, with $\Sigma =\bigcup _{v\in \mathcal {V}}\Sigma _v$, such that the following holds:

1. Every $\Sigma _v$ is semi-open in Σ with respect to the non-uniform topology induced by $d_{\textrm{nu}}$.
2. If execution $\gamma \in \Sigma$ is *v*-valent, then $\gamma \in \Sigma _v$.

### Proof

(⇒): Assume stabilizing consensus is solvable with a proper decision function Δ, and define $\Sigma _v = \Delta ^{-1}(v)$. Since Δ is semi-continuous by Lemma 5.3, it follows from Definition 3.10 that $\Sigma _v$ is semi-open, as asserted in (1). Validity immediately implies property (2).

(⇐): Assuming that every $\Sigma _v$ is semi-open and contains all *v*-valent executions, we define a proper stabilizing consensus protocol with weak validity by defining the decision functions $\Delta _p:\mathcal {C}\rightarrow \mathcal {V}$, $p\in \Pi$ according to Table 1.

Let us assume that the actual run is the admissible execution $\gamma =(C^t)_{t\ge 0}\in \Sigma$, which is of course unknown to the process $p \in {{\,\textrm{Ob}\,}}(\gamma )$. However, *p* can observe the sequence of local views $(V_p(C^{t_i}))_{t_i\ge 0}$, where $t_0=0$ and $t_i$ denotes the (unknown) global time of the *i*-th computing step of *p*. Considering the set of indistinguishable admissible executions according to Eq. (6), we define *p*’s decision function for configuration $C^{t_i}$ at time ${t_i}$ in γ as shown in Eq. (8)–Eq. (11) in Table 1, where we use ${{\,\mathrm{\partial }\,}}{{\,\mathrm{\partial in}\,}}\Sigma _v$ to denote the boundary of the included boundary ${{\,\mathrm{\partial in}\,}}\Sigma _v$ (taken in the subspace $Y=\bigcup _{w\in \mathcal {V}} {{\,\mathrm{\partial in}\,}}\Sigma _w$). The function $\Delta _p$ is well defined, since the decision sets $\Sigma _v$ and hence also ${{\,\mathrm{\partial in}\,}}\Sigma _v$ are pairwise disjoint, $p\in {{\,\textrm{Ob}\,}}(\gamma )$, and $\gamma \in D_p(\gamma ,{t_i})$ by Lemma 5.6.(ii).

**Fig. 4:**
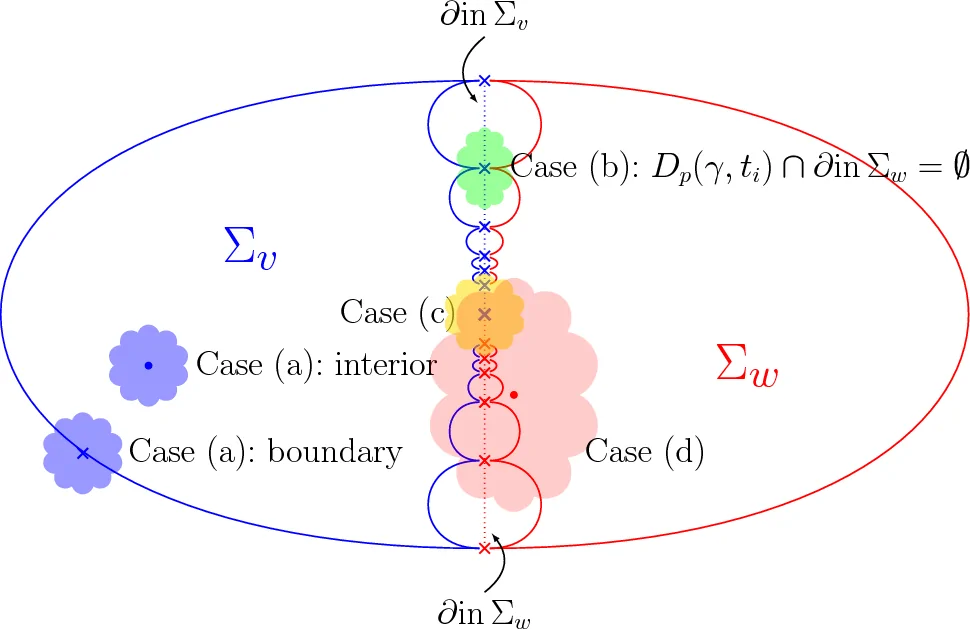
Illustration of the two cases in the proof of Theorem 5.7, depicting the four different cases for the decision function $\Delta _p(C^{t_i})$. The blue, green, yellow, and red cloud represents $D_p(\gamma ,t^i)$ for Case (a), (b), (c), and (d), respectively. The included boundaries ${{\,\mathrm{\partial in}\,}}\Sigma _v$ (resp. ${{\,\mathrm{\partial in}\,}}\Sigma _w$) are formed by the limit points (marked by a cross) that lie on the blue (resp. red) dotted lines. The thick limit point at the center of the yellow cloud lies in the intersection of ${{\,\mathrm{\partial }\,}}{{\,\mathrm{\partial in}\,}}\Sigma _v$ and ${{\,\mathrm{\partial }\,}}{{\,\mathrm{\partial in}\,}}\Sigma _w$; we assume here w.l.o.g. that it belongs to ${{\,\mathrm{\partial in}\,}}\Sigma _v$ (blue).

To show Stabilizing Agreement (SA), we need to distinguish several cases, which are all illustrated in Fig. 4. Regarding γ with $p\in {{\,\textrm{Ob}\,}}(\gamma )$, there are two possibilities:

(1) $\gamma \in {{\,\textrm{Int}\,}}(\Sigma _v)$ is an interior point. Then, Eq. (8) (Case (a): interior point, see Fig. 4) in conjunction with Lemma 5.6.(iii) immediately guarantees $\Delta _p(C^{t_i}) = v$ for all ${t_i}\ge L$, i.e., process *p* stabilizes on the decision value *v* by time *L* in execution γ. To also show that all obedient processes decide the same value *v* in $\gamma \in {{\,\textrm{Int}\,}}(\Sigma _v)$, we assume for a contradiction that process $q \in {{\,\textrm{Ob}\,}}(\gamma )$ decides value w≠v in configuration $C^{t_i}$ in execution γ. But then, by the definition of the function $\Delta _q$, we must have $\gamma \in D_q(\gamma ,{t_i})=\bigl \{\delta =(D^t)_{t\ge 0} \in \Sigma \mid (q \in {{\,\textrm{Ob}\,}}(\delta )) \wedge \exists s: C^{t_i} \sim _p D^s\bigr \} \subseteq \Sigma _w$. But this is impossible, since $\gamma \in \Sigma _v$ and $\Sigma _v\cap \Sigma _w = \emptyset$.

(2) $\gamma \in {{\,\mathrm{\partial in}\,}}\Sigma _v$ is an included boundary point, and hence a limit point of $\Sigma _v$ by Definition 5.1. If $\gamma \not \in {{\,\mathrm{\partial }\,}}\Sigma _w$ for any w≠v, then there will be some time ${t_i}$ from which on $D_p(\gamma ,{t_i})\subseteq \Sigma _v$ holds. Exactly as for case (1) above, Lemma 5.6.(iii) implies (SA) according to Eq. (8) (Case (a): boundary point, see Fig. 4) here. Otherwise, $D_p(\gamma ,{t_i})$, considered as a neighborhood of γ in the sense that $\gamma \in D_p(\gamma ,{t_i})$ by Lemma 5.6.(ii), intersects not ony $\Sigma _v$ (as $\gamma \in \Sigma _v$ by assumption) but also in one or more other decision sets $\Sigma _w$, since γ is also a limit point of those.

In order to determine the (a priori unknown) decision set $\Sigma _v$ containing γ, $\Delta _p$ considers the set $D_p(\gamma ,{t_i})\cap {{\,\mathrm{\partial in}\,}}\Sigma _w$ of candidate limit points in $\Sigma _w$, for all *w* where this candidate set is non-empty. We need to distinguish two subcases here:

(b) If γ is a limit point of the executions in ${{\,\mathrm{\partial in}\,}}\Sigma _v$, i.e., $\gamma \in {{\,\mathrm{\partial }\,}}{{\,\mathrm{\partial in}\,}}\Sigma _v$, but not also a limit point of any other ${{\,\mathrm{\partial in}\,}}\Sigma _w$, i.e., $\gamma \not \in {{\,\mathrm{\partial }\,}}{{\,\mathrm{\partial in}\,}}\Sigma _w$, there will be a time ${t_i}$ after which $D_p(\gamma ,{t_i})\cap {{\,\mathrm{\partial in}\,}}\Sigma _w=\emptyset$. Since of course $\gamma \in D_p(\gamma ,{t_i})\cap {{\,\mathrm{\partial in}\,}}\Sigma _v$, Eq. (9) (Case (b), see Fig. 4) secures (SA) according to Lemma 5.6.(iii) again.

(c) If there is at least one other candidate set for *w*, besides the one for *v*, for which γ is also a limit point of the limit executions in ${{\,\mathrm{\partial in}\,}}\Sigma _w$, more specifically, if there is a sequence of limit executions $\alpha _1,\alpha _2, \dots \in {{\,\mathrm{\partial in}\,}}\Sigma _w$ with $\alpha _i \in B_{2^{-i}}(\alpha )$ for some limit point $\alpha \in {{\,\mathrm{\partial }\,}}{{\,\mathrm{\partial in}\,}}\Sigma _w$, in addition to some sequence $\gamma _1,\gamma _2,\dots \in {{\,\mathrm{\partial in}\,}}\Sigma _v$ with $\gamma _i \in B_{2^{-i}}(\gamma )$ with a limit point $\gamma \in {{\,\mathrm{\partial }\,}}{{\,\mathrm{\partial in}\,}}\Sigma _v$ satisfying $d_{\textrm{nu}}(\alpha ,\gamma )=0$, then the fact that $\gamma \in \Sigma _v$ also forces $\alpha \in \Sigma _v$ and hence $\alpha \in {{\,\mathrm{\partial in}\,}}\Sigma _v$: After all, the decision value of α and γ must be the same since $d_{\textrm{nu}}(\alpha ,\gamma )=0$. Consequently, *v* is uniquely determined by the decision set $\Sigma _v$ that contains all limits, so deciding *v* according to Eq. (10) (Case (c), see Fig. 4) ensures (SA) also in this case.

To show that all obedient processes decide the same value *v* also here, we again assume for a contradiction that process $q \in {{\,\textrm{Ob}\,}}(\gamma )$ decides value w≠v in configuration $C^{t_i}$ in execution $\gamma \in \Sigma _v$ by one of the cases in (2). By the definition of the function $\Delta _q$, we must have $\gamma \in D_q(\gamma ,{t_i})$. Since any of these cases requires $\gamma \in \Sigma _w$, this is impossible since $\gamma \in \Sigma _v$ and $\Sigma _v\cap \Sigma _w = \emptyset$.

If none of the above Cases (a)–(c) applies, $D_p(\gamma ,{t_i})$ is still too large to uniquely determine the decision set γ belongs to. In that case, any decision value according to Eq. (11) (Case (d), see Fig. 4) could be picked by $\Delta _p$.

Finally, Validity (V) immediately follows from property (2). □

We conclude this section with Lemma 5.8, which shows that non-proper stabilizing consensus protocols can indeed be turned into proper ones:

### Theorem 5.8

(Reduction of non-proper to proper). Any decision function $\Delta^{\prime }$ of a non-proper stabilizing consensus protocol that satisfies the IAIA condition can be turned into a decision function Δ that also satisfies Definition 5.1.

### Proof

To construct Δ, we set $\Delta (\gamma )=\Delta '(\gamma )$ for all executions γ not violating properness according to Definition 5.1. For every γ that violates properness, the following change is applied: Since $\gamma \in {{\,\mathrm{\partial in}\,}}\Sigma _v$ is not an isolated point in Σ, it must satisfy $\gamma \in {{\,\mathrm{\partial }\,}}\Sigma _w$ for at least one w≠v. We pick any such *w* and define $\Delta (\gamma )=w$, i.e., we change the original decision value of every such execution γ from *v* to *w*. Note that this amounts to moving γ from ${{\,\mathrm{\partial in}\,}}\Sigma _v$ to ${{\,\mathrm{\partial in}\,}}\Sigma _w$, and thus from $\Sigma _v$ to $\Sigma _w$.

Whereas this change cannot invalidate stabilizing agreement (SA) provided by the original $\Delta^{\prime }$, we need to make sure that changing the decision value of γ from *v* to *w* does not violate validity (V). This is impossible, however, as such a violation could only happen if γ was *v*-valent, in which case $$\gamma \in {{\,\mathrm{\partial }\,}}\Sigma _w$$ could not have occured. □

## Equivalence of stabilizing consensus with weak and strong validity

A byproduct of the topological characterization for terminating consensus developed in [32] is the equivalence of weak validity (V) and strong validity (SV) (recall Definition 2.1). For binary consensus, i.e., $|\mathcal {V}|=2$, this is a well-known fact [41, Ex. 5.1], for larger input sets, it was, to the best of our knowledge, not known before [32]. In this section, we will show that this equivalence is also true for stabilizing consensus.

We start with some definitions needed for formalizing this condition:

### Definition 6.1

*(Heard-of sets* [32, Def. 7.1]*).* For every process $p\in \Pi$, there is a function $HO_p:\mathcal {C}\rightarrow 2^\Pi$ that maps a configuration $C\in \mathcal {C}$ to the set of processes $HO_p(C)$ that *p* has ever heard of in *C*. Its extension to execution $\gamma =(C^t)_{t\ge 0}$ is defined as $HO_p(\gamma ) = \bigcup _{t\ge 0} HO_p(C^t)$.

Heard-of sets have the following obvious properties: For executions $\gamma = (C^t)_{t\ge 0}$, $\delta = (D^t)_{t\ge 0}$ and all t≥0,

(i) $$p\in HO_p(C^t)$$, and $$HO_p(C^t) = HO_p(D^t)$$ if $$C^t \sim _p D^t$$,
(ii) $$HO_p(C^t)\subseteq HO_p(C^{t+1})$$,
(iii) for all $x\in \Pi$, if $x\in HO_q(C^t) \cap HO_q(D^t)$ and $C^t \sim _q D^t$, then $I_x(\gamma ) = I_x(\delta )$ (where $I_p(\gamma )$ denotes the initial value of process *p* in execution γ).

Note that a process *p* may have heard of some other process *q* indirectly, i.e., transitively via multi-hop communication, in the sense that some other process *r* who has heard-of *q* at some earlier time tells this to *p* later.

The additional *independent arbitrary input assignment* condition (abbreviated *IAIA condition*) stated in Definition 6.2 secures (i) that for every execution γ with initial value assignment I(γ), there is a an isomorphic execution δ w.r.t. the HO sets of all processes that starts from an arbitrary other initial value assignment I(δ), and (ii) all initial value assignments with elements taken from the set $\mathcal {V}$ are possible. Whereas all stabilizing consensus algorithms we are aware of satisfy this condition, there might be exceptions, as the condition-based terminating consensus algorithms proposed in [37] reveal.

### Definition 6.2

*(Independent arbitrary input assignment (IAIA) condition* [32, Def. 7.2]*).* Let $I:\Pi \rightarrow \mathcal {V}$ be some assignment of initial values to the processes, and $\Sigma ^{(I)}\subseteq \Sigma$ be the set of admissible executions with that initial value assignment. We say that Σ satisfies the *independent input assignment* condition, if and only if for any two assignments *I* and *J*, we have $\Sigma ^{(I)} \cong \Sigma ^{(J)}$, that is, there is a bijective mapping $f_{I,J}:\Sigma ^{(I)}\rightarrow \Sigma ^{(J)}$ such that for all $\gamma = (C^t)_{t\ge 0} \in \Sigma ^{(I)}$ and $\delta = (D^t)_{t\ge 0} \in \Sigma ^{(I)}$, writing $f_{I,J}(\gamma ) = (C_f^t)_{t\ge 0}$ and $f_{I,J}(\delta ) = (D_f^t)_{t\ge 0}$, the following holds for all t≥0 and all $p\in \Pi$:

1. $${{\,\textrm{Ob}\,}}(C^t) = {{\,\textrm{Ob}\,}}(C_f^t)$$
2. $C^t \sim _p D^t$ if and only if $C_f^t \sim _p D_f^t$
3. $$HO_p(C^t) = HO_p(C_f^t)$$
4. $C^t \sim _p C_f^t$ if $I_q = J_q$ for all $q\in HO_p(C^t)$

We say that Σ satisfies the *independent arbitrary input assignment condition* (IAIA condition), if it satisfies the independent input assignment condition for every choice of $I:\Pi \rightarrow \mathcal {V}$.

Lemma 6.4 below will reveal that if proper stabilizing consensus satisfying the IAIA condition (with weak validity) is solvable, then every connected component in $\Sigma _v$ needs to be broadcastable.

### Definition 6.3

*(Broadcastability* [32, Def. 7.3]*).* We call a subset $A \subseteq \Sigma$ of admissible executions *broadcastable* by the broadcaster $p\in \Pi$, if, in every execution $\gamma \in A$, every obedient process $q\in {{\,\textrm{Ob}\,}}(\gamma )$ eventually hears from process *p*, i.e., $p\in HO_q(\gamma )$, and hence knows [i.e., has received] $I_p(\gamma )$.

### Lemma 6.4

(Broadcastable connected components). A connected component $\Sigma _\gamma$ of any decision set $\Sigma _v$ for proper stabilizing consensus satisfying the IAIA condition (where γ can be any execution contained in the connected component), which is not broadcastable for any process, contains *w*-valent executions, for every $w\in \mathcal {V}$.

### Proof

To prove our lemma, we consider the finite sequence of executions $\gamma =\alpha _0,\alpha _1,\dots ,\alpha _n=\gamma _w$ obtained from γ by changing the initial values of the processes 1,⋯,n in I(γ) to an arbitrary but fixed *w*, one by one (it is here where we need the IAIA condition). We show by induction that $\alpha _p \in \Sigma _{\gamma }$ for every $p\in \{0,\dots ,n\}$, which proves our claim since $\alpha _n=\gamma _w$ is *w*-valent.

The induction basis p=0 is trivial, so suppose $\alpha _{p-1}\in \Sigma _{\gamma }$ according to the induction hypothesis. If it happens that $I_p(\alpha _{p-1})=I_{p}(\gamma )=w$ already, nothing needs to be done and we just set $\alpha _{p}=\alpha _{p-1} \in \Sigma _{\gamma }$. Otherwise, $\alpha _{p}$ is $\alpha _{p-1}$ with the initial value $I_{p}(\alpha _{p})$ changed to *w*. Now suppose for a contradiction that $\alpha _{p} \in \Sigma _{\alpha _{p}} \ne \Sigma _{\gamma }$.

Since $$\Sigma _{\gamma }$$ is not broadcastable by any process, hence also not by *p*, there is some execution $$\eta \in \Sigma _{\gamma }$$ with $$\eta = (C^t)_{t\ge 0}$$ and a process q≠p with $$q \in {{\,\textrm{Ob}\,}}(C^t)$$ and the initial value $$I_p(\eta )$$ not in *q*’s view $$V_{q}(C^t)$$ for every t≥0. Thanks to the IAIA condition in Definition 6.2, there is also an execution $$\delta =f_{I(\eta ),I'}(\eta ) \in \Sigma _{\alpha _{p}}$$ that matches η, i.e., is the same as η except that $I\left(\delta \right)=I^{\prime }$ with $$I'_q=I_q(\eta )$$ for $$p \ne q \in \Pi $$ but possibly $$I'_p\ne I_p(\eta )$$. It follows that $$d_{q}(\eta ,\delta )=0$$ with $$q\in {{\,\textrm{Ob}\,}}(\eta )\cap {{\,\textrm{Ob}\,}}(\delta )$$ and hence $$d_{\textrm{nu}}(\eta ,\delta )=0$$. Consequently, $$\delta \in \Sigma _{\gamma }$$ and hence $$\Sigma _{\alpha _{p}}=\Sigma _{\gamma }$$, which provides the required contradiction and completes the induction step. □

As a consequence of Lemma 6.4, it turns out that *any* connected broadcastable set has a diameter strictly smaller than 1 in our non-uniform topology.

### Definition 6.5

*(Diameter of a set).* For $A \subseteq \mathcal {C}^{\omega }$, depending on the distance function *d* that induces the desired topology, define *A*’s diameter as $d(A)=\sup \{d(\gamma ,\delta )\mid \gamma , \delta \in A\}$.

### Lemma 6.6

(Diameter of broadcastable connected sets [32, Lem. 7.6]). If a connected set $A\subseteq \Sigma$ of admissible executions is broadcastable by some process *p*, then $d_{\textrm{u}}(A) \le d_{p}(A)\le 1/2$, as well as $d_{\textrm{nu}}(A) \le 1/2$, i.e., *p*’s initial value satisfies $I_p(\gamma )=I_p(\delta )$ for all $\gamma ,\delta \in A$.

### Proof

Our proof below for $d_p(A)\le 1/2$ translates literally to any $d \in \{d_p, d_{\textrm{u}}, d_{\textrm{nu}}\}$; the statement $d_{\textrm{u}}(A) \le d_{p}(A)$ follows from the definition in Eq. (4).

Broadcastability by *p* implies that, for any $\gamma \in A$ with $\gamma =(C^t)_{t\ge 0}$, every process *q* has $I_p(\gamma )$ in its local view $V_{q}(C^{T(\gamma )})$ for some $0<T(\gamma )<\infty$ or is not obedient any more. Abbreviating t=T(γ), consider any $\delta \in B_{2^{-t}}(\gamma ) \cap A$ with $\delta = (D^t)_{t\ge 0}$. By definition of $B_{2^{-t}}(\gamma )$, there must be some process $q \in {{\,\textrm{Ob}\,}}(D^t)\cap {{\,\textrm{Ob}\,}}(C^t)$ with $V_{q}(D^{t})=V_{q}(C^{t})$. Definition 6.1.(iii) thus guarantees $I_p(\delta )=I_p(\gamma )$.

We show now that $I_p(\delta )=I_p(\gamma )$ can be asserted for *every* $\delta \in A$ where *p* is obedient. For a contradiction, suppose that this is not the case and let U(γ) be the union of the balls recursively defined as follows: $U_0(\gamma )=\{\gamma \}$, for m>0, $U_m(\gamma ) = \bigcup _{\delta \in U_{m-1}(\gamma )} (B_{2^{-T(\delta )}}(\delta ) \cap A)$, and finally $U(\gamma )=\bigcup _{m\ge 0} U_m(\gamma )$. As a union of open balls intersected with *A*, which are all open in *A*, both $U_m(\gamma )$ for every m>0 and U(γ) is hence open in *A*. For every $\delta \in A\setminus U(\gamma )$, U(δ) is also open in *A*, and so is $V(\gamma )=\bigcup _{\delta \in A\setminus U(\gamma )}U(\delta )$. However, the open sets U(γ) and V(γ) must satisfy $U(\gamma ) \cap V(\gamma ) = \emptyset$ (as they would be the same otherwise) and $U(\gamma )\cup V(\gamma )=A$, hence *A* cannot be connected. □

Lemma 6.4 in conjunction with Lemma 6.6 immediately imply:

### Corollary 6.7

(Broadcastable $\Sigma _{\gamma }$). If proper stabilizing consensus with weak validity is solvable, then every connected component $\Sigma _{\gamma }\subseteq \Sigma _v$ must be broadcastable by some process *p*. In every execution $\gamma '\in \Sigma _{\gamma }$, the broadcaster *p* has the same initial value $I_p(\gamma ')$ (and, since $\Sigma _\gamma$ is a connected component of $\Sigma _v$, the same decision value *v*).

### Proof

Lemma 6.4 reveals that every connected component $\Sigma _\gamma$ of any $\Sigma _v$ must be broadcastable by some process *p*, whereas Lemma 6.6 proves that *p* must have the same initial value in every execution in $\Sigma _\gamma$. □

To emphasize the key role of Corollary 6.7 for the equivalence of weak validity (V) and strong validity (SV), note that the transition from (V) to (SV) in Theorem 5.7 just requires the replacement of condition (2), i.e., *“If execution* $\gamma \in \Sigma$ *is* *v*-*valent, then* $\gamma \in \Sigma _v$”, by *“If execution* $\gamma \in \Sigma _v$, *then there is a process* *p* *with initial value* $I_p(\gamma )=v$”. This change results in a strong version of our theorem, since this modification is completely transparent for the proof of Theorem 5.7: in this proof, the definition of (V) is just copied to the characterization and vice versa. Note also that (V) and (SV) are equivalent for *v*-valent executions.

The crucial role of Corollary 6.7 is that it *always* allows to turn a proper weak stabilizing consensus protocol into a strong one, as it reveals that if proper weak stabilizing consensus with the IAIA condition is solvable, then every connected component $\Sigma _\gamma \subseteq \Sigma _v$ must have at least one common broadcaster $b=b(\gamma ')=b(\Sigma _\gamma )$ that has the same initial value $I_b(\gamma ')=I_b(\gamma )$ in all executions $\gamma '\in \Sigma _\gamma$. Herein, we abuse the notation $b(\Sigma _\gamma )$ to define a *broadcaster map* for connected components, which returns a single process that is broadcaster in every execution contained in $\Sigma _\gamma$. Whereas $b(\sigma _\gamma )$ need not be uniquely definable, since several processes could be broadcasters in all executions in $\Sigma _\gamma$, any choice will do. Note carefully, however, that $I_{b(\Sigma _\gamma )}(\gamma )$ could be *different* from *v* for a connected component $\sigma _\gamma \subseteq \Sigma _v$.

Anyway, if decision sets exist that allow to solve consensus with weak validity according to Theorem 5.7, we will prove in Theorem 6.9 below that one can reshuffle the connected components among the decision sets to form *strong* decision sets, which use the initial value of some broadcaster for assigning a connected component to a decision set:

### Definition 6.8

*(Strong decision sets).* Let Σ be the set of admissible executions of any (weak or strong) proper stabilizing consensus protocol satisfying the IAIA condition. A *strong decision set* $\Sigma _v$ for $v\in \mathcal {V}$ satisfies

12 $$\begin{aligned} \Sigma _v = \bigcup _{p \in \Pi } \Sigma _v^p \qquad \text{ with }\qquad \Sigma _v^p = \bigcup _{\begin{array}{c} \gamma \in \Sigma \\ b(\Sigma _\gamma )=p \\ I_{p}(\gamma )=v \end{array}} \Sigma _\gamma . \end{aligned}$$

Note that strong decision sets need not be unique, as $$b(\Sigma _\gamma )$$ need not be unique. The canonical choice to make it uniquely defined would be to take the lexically smallest $$p=b(\Sigma _\gamma )$$ among all broadcasters $p^{\prime }\geq p$ in $$\Sigma _\gamma $$.

Practically, all that needs to be done for a connected component $\Sigma _\gamma \subseteq \Sigma _v$ that only has a broadcaster *p* with initial value $I_p(\gamma )=w\ne v$ in every execution γ is to change the decision value from *v* to *w*, which will automatically move $\Sigma _\gamma$ from $\Sigma _v$ to $\Sigma _w$. The following Theorem 6.9 proves that this is indeed always feasible:

### Theorem 6.9

(Equivalence of weak and strong validity). Proper stabilizing consensus satisfying the IAIA condition with weak validity is solvable in a model if and only if consensus satisfying the IAIA condition with strong validity is solvable in this model.

### Proof

Since strong validity also implies weak validity, we only need to prove the other direction. So assume that $\Sigma _v$, $v\in \mathcal {V}$, are the decision sets of a proper stabilizing consensus protocol that satisfies the IAIA condition with weak validity. By reshuffling their connected components appropriately, we will construct a strong decision set.

Theorem 5.7 guarantees that every $\Sigma _v$ is semi-open and contains all *v*-valent executions. According to Corollary 6.7, we can decompose $\Sigma _v$ as follows:

$$\begin{aligned} \Sigma _v = \bigcup _{p \in \Pi } \Sigma _v^p \qquad \text{ with }\qquad \Sigma _v^p = \bigcup _{\begin{array}{c} \gamma \in \Sigma _v\\ b(\Sigma _\gamma )=p \end{array}} \Sigma _\gamma , \end{aligned}$$

where $\Sigma _\gamma$ is the connected component of $\Sigma _v$ that contains γ and $b(\Sigma _\gamma )$ is any broadcasting map. Obviously, $\Sigma _v$, $v\in \mathcal {V}$ is usually *not* a strong decision set, since $\Sigma _v^p$ may contain a connected component $\Sigma _\gamma$ satisfying $I_{b(\Sigma _\gamma )}(\gamma )=w \ne v$.

We will now argue that we can move any such $\Sigma _\gamma$ from $\Sigma _v^p$ to $\Sigma _w^p$ without destroying (i) semi-openness and (ii) properness of the resulting decision sets, which comprise $\Sigma _w^p \cup \Sigma _\gamma$ and $\Sigma _v^p \setminus \Sigma _\gamma$ after this move. Recall that moving $\Sigma _\gamma$ means changing the decision value of all its executions from *v* to *w*.

Regarding (i), it follows from Theorem 3.9 that every connected component of a semi-open set is semi-open, since every connected component is closed. $\Sigma _\gamma$ is hence semi-open, such that $\Sigma _w^p \cup \Sigma _\gamma$ is also semi-open by Theorem 3.4. Similarly, removing $\Sigma _\gamma$ from $\Sigma _v^p$ cannot invalidate semi-openness either.

Regarding (ii), we need to show that adding $\Sigma _\gamma$ to $\Sigma _w^p$ does not add an execution δ that is not a limit point in $\Sigma _w^p \cup \Sigma _\gamma$ unless δ is an isolated point in Σ. Clearly, since $\Sigma _\gamma$ is a connected component of $\Sigma _v$, it is closed and satisfies the properness condition. As it thus contains all its limit points as proper limits of executions in $\Sigma _\gamma$, moving $\Sigma _\gamma$ to $\Sigma _w$ cannot invalidate properness of the latter. □

We conclude this section with the remark that the practical utility of our equivalence appears to be limited: Since our universal stabilizing consensus algorithm of Theorem 5.7 depends on the a priori knowledge of the decision sets, it does not give any clue on how to develop a practical strong consensus protocol from a practical weak consensus protocol in a given model. In fact, determining and agreeing upon a broadcaster in executions of a stabilizing consensus algorithm that are not *v*-valent *just based on the local views of the processes* sounds like a hard algorithmic problem. Unsurprisingly, we are not aware of any attempt on solving this problem in the existing literature.

## Applications

In this section, we will apply our findings to the few existing results on stabilizing consensus. This way, we will provide a topological explanation of why the problem is solvable/impossible in certain models.

### Stabilizing consensus in asynchronous crash-prone systems

We start with the simple stabilizing consensus protocol for crash-prone asynchronous systems proposed in [4]. It is designed for any number f<n of crashes, and processes that are connected by fair-lossy point-to-point links. We recall that fair-lossy links only provide the guarantee that sending an infinite number of messages also causes an infinite number of messages to be received.

The protocol works as follows: In every step, every process *p* broadcasts its current decision value $O_p$. If a message containing value *v* has been received from any process, *p* sets $O_p = \min \{O_p,v\}$. Note that the processes execute their algorithm without any synchronization, and there is no reference to configurations in the analysis of this protocol: when, say, process *p* makes a step and sets $O_p$, one does not care what $O_q$ is in the current configuration. Consequently, we can just take $\chi _p(t)=t$, i.e., global real-time, in our topological analysis.

The above protocol gives raise to (strong) decision sets $\Sigma _v$, $v\in \mathcal {V}$, defined by $\Sigma _v = \bigl \{\gamma \in \Sigma \mid \min _{p \in bc(\gamma )}\{I_p(\gamma )\}=v\bigr \}$, where $bc(\gamma )=\bigl \{p\mid p \in \bigcap _{q \in {{\,\textrm{Ob}\,}}(\gamma )}HO_q(\gamma )\bigr \}$ denotes the set of broadcasters in γ. Note that the processes in bc(γ) need not necessarily be obedient.

To show that every $\Sigma _v$ is semi-open, we need to verify that every $\gamma \in \Sigma _v$ is either (i) an interior point ($\gamma \in {{\,\textrm{Int}\,}}(\Sigma _v)$) or else (ii) a point in the included boundary ($\gamma \in {{\,\mathrm{\partial in}\,}}\Sigma _v$). Writing $\gamma =(C^t)_{t\ge 0}$, $\gamma \in \Sigma _v$ implies that there is some $p\in bc(\gamma )$ with $I_p(\gamma )=v$ and a time *t* such that $p \in HO_q(C^t)$ for every $q\in {{\,\textrm{Ob}\,}}(\gamma )$. Fix some *t* where this holds for every process $p\in bc(\gamma )$ with $I_p(\gamma )=v$ (there may be several), and assume that there is some $\delta =(D^t)_{t\ge 0} \in B_{2^{-t}}(\gamma ) \cap \Sigma _w$, where we first assume w>v. By the definition of $d_{\textrm{nu}}$ (Eq. (5)), there must hence be some process $s \in {{\,\textrm{Ob}\,}}(\gamma )\cap {{\,\textrm{Ob}\,}}(\delta )$ that has the same view $V_s(D^t)=V_s(C^t)$ at time *t*. This implies, however, that $p\in HO_s(\delta )$, so *s* also knows *v*. Since $s\in {{\,\textrm{Ob}\,}}(\delta )$, *s* will eventually broadcast *v* to all obedient processes in δ (recall the fair-lossy link assumption), which reveals that $p \in bc(\delta )$ as well. Consequently, no such δ can exist, which results in $B_{2^{-t}}(\gamma ) \subseteq \Sigma _v$ and thus $\gamma \in {{\,\textrm{Int}\,}}(\Sigma _v)$ according to (i).

However, in the argument above, we might observe $$\delta \in B_{2^{-t}}(\gamma ) \cap \Sigma _w$$ for some w<v. In this case, the existence of δ would not be contradictory as before. Actually, there are two possibilities here: If there is some finite $t^{\prime }>t$ such that $$B_{2^{-t'}}(\gamma ) \cap \Sigma _w=\emptyset $$, we find $$B_{2^{-t'}}(\gamma ) \subseteq \Sigma _v$$, which confirms $$\gamma \in {{\,\textrm{Int}\,}}{\Sigma _v}$$. If, however, an “offending” $$\delta _i' \in B_{2^{-i}}(\gamma ) \cap \Sigma _w$$ can be found for every i≥t, then $$\gamma = \lim _{i\rightarrow \infty } \delta _i' \in {{\,\mathrm{\partial }\,}}\Sigma _w$$ is a limit point of $$\Sigma _w$$. Obviously, none of the broadcasters $$p' \in bc(\delta _i')$$ with $$I_{p'}(\delta _i')=w<v$$ can also satisfy $$p' \in bc(\gamma )$$. The only way how a sequence of $$\delta _1',\delta _2',\dots \in \Sigma _w$$ can converge to $$\gamma \in \Sigma _v$$ is hence when *all* the obedient processes in $$\delta _i'$$ hear of the value *w* broadcast by $p^{\prime }$ not before or at time *i*: Since $$\delta _i' \in B_{2^{-i}}(\gamma )$$, no process that is obedient in both γ and $$\delta _i'$$ can have heared from $p^{\prime }$. This, in turn, is only possible if $p^{\prime }$ is not obedient in γ, in a way that it never tells any obedient process about *w*. But this implies that the sequence $$\delta _1,\delta _2, \dots $$, which is the same as $$\delta _1',\delta _2',\dots $$ except that also $p^{\prime }$ has the initial value $$I_{p'}(\delta _i)=v$$, satisfies $$\delta _i \in \Sigma _v$$ and converges to γ as well. Consequently, $$\gamma \in {{\,\mathrm{\partial in}\,}}\Sigma _v$$ is a limit point according to case (ii).

Therefore, all $\Sigma _v$ are indeed strong and semi-open.

### Stabilizing consensus in synchronous systems controlled by a message adversary

We next turn our attention to synchronous dynamic networks controlled by a message adversary [29]. We will provide topological explanations of the (few) core results on stabilizing consensus established in the past [5, 22, 23]. Most of these results are from [5] or are based on the MinMax algorithm introduced there.

The general setting is a lock-step synchronous system of *n* fault-free processes Π, so ${{\,\textrm{Ob}\,}}(\gamma )=\Pi$ for every $\gamma =(C^t)_{t\ge 0} \in \Sigma$ and $\chi _p(C^t)=t$ for all $p \in \Pi$. The communication in every round is controlled by a message adversary, which determines the sequence of communication graphs $\mathcal {G}=(\mathcal {G}^t)_{t\ge 1}$, subsequently called *communication pattern*, that governs execution γ. A message adversary can be identified with the set of admissible communication patterns it allows. Since all our protocols are deterministic, $\mathcal {G}$ and the input assignment I(γ) uniquely determines γ. Note that we implicitly assume that every communication graph always contains all self-loops p→p for $p \in \Pi$.

#### Lossy-link model possibility

We start with stabilizing consensus in the lossy-link model [24–27], which is arguably the most prominent example of a message adversary. The underlying system consists of only two processes $\Pi =\{l,r\}$ (the “left” and the “right” process), where the communication graph in every round of the communication pattern $\mathcal {G}=(\mathcal {G}^t)_{t\ge 1}$ of an execution $\gamma =(C^t)_{t\ge 0}$ is taken from the set $LL=\{l \leftarrow r, l \leftrightarrow r, l \rightarrow r\}$. For conciseness, we will abbreviate $LL= \{\leftarrow , \leftrightarrow , \rightarrow \}$, with the implicit meaning $\leftarrow \; = \; l \leftarrow r$ etc., and write $\mathcal {G}\in LL^{\omega }$ in the sequel.

The LL model is the most prominent example of an *oblivious* message adversary [27]. It is well-known, see e.g. [24–27], that terminating consensus is impossible here. Stabilizing consensus can be solved in the LL model, however, by using a simple MinMax algorithm: Translating the notation from [23] to our setting, the decision function of process *p* (computed at the end of round t≥1 in $\gamma =(C^t)_{t\ge 0}$) can be expressed as

13 $$\begin{aligned} \Delta _p(C^t) = \max _{q:V_q(C^{t-1}) \in V_p(C^t)} \left\{ \min _{s \in HO_q(C^{t-1})}I_s(\gamma ) \right\} . \end{aligned}$$

To correctly interprete this definition, note that *p*’s view $V_p(C^t)$ is the (in the case of anonymous processes, disjoint) union of the views $V_q(C^{t-1})$ that process *p* receives from the processes (including q=p) in round *t*. $HO_q(C^{t-1})$ is the set of all processes that *q* has heard-of in $V_q(C^{t-1})$.

It is easy to see that this $\Delta _p$ solves stabilizing consensus in every $\gamma \in \Sigma$. Indeed, the only non-trivial case is when $I_l(\gamma )\ne I_r(\gamma )$: Assuming w.l.o.g. $m=I_l(\gamma ) < I_r(\gamma )=m'$, if *r* receives a message from *l* in some round t<∞ for the first time, it sets $O_r=m$ at the end of round *t*. Moreover, $O_l=m$ is set by process *l* at the end of round *t* if it does not receive a message from *r* in round *t*, and in round t+1 otherwise, irrespectively of whether it gets a message from *r* in round t+1 or not. If, on the other hand, *r* never receives such a message, $HO_r(C^t)=\{r\}$ for all t≥0. Hence, $O_r=m'$ from t=0 on, and since the maximum view found in $\Delta _l(C^t)$ is the one of q=r, $O_l=m'$ from round t=1 on as well.

As in Section 7.1, this protocol gives raise to strong decision sets $\Sigma _v$, $v\in \mathcal {V}$, defined by $\Sigma _v = \bigl \{\gamma \in \Sigma \mid \min _{p \in bc(\gamma )}\{I_p(\gamma )\}=v\bigr \}$, where $bc(\gamma )=\bigl \{p\mid p \in \bigcap _{q \in \Pi }HO_q(\gamma )\bigr \}$ denotes the set of broadcasters in γ. The argument is essentially the same as in Section 7.1, except that $\delta _i'\in \Sigma _w$ in the sequence $\delta _1',\delta _2', \dots \rightarrow \gamma \in \Sigma _v$ lets the first message conveying the smaller input value w<v to the other process arrive at time *i* (i.e., in round *i*). Note that the only boundary points can be executions γ where either $I_l(\gamma ) < I_r(\gamma )$ and $\mathcal {G}= \{\leftarrow \}^\omega$ or else $I_l(\gamma ) > I_r(\gamma )$ and $\mathcal {G}= \{\rightarrow \}^\omega$ here, since otherwise the broadcaster with initial value w<v would be a broadcaster in $\gamma \in \Sigma _v$, which is impossible.

Obviously, the LL model considered above allows to solve stabilizing consensus via the MinMax algorithm in Definition 13 only if both processes are active from $t_r^a=t_l^a=0$ on. To also cover $t_r^a >0$ and/or $t_l^a > 0$ allowed by the system model of Section 2, $\diamond LL = \{\text {---}\}^*LL^{\omega }$, which allows an arbitrary finite prefix of empty graphs before the LL suffix, is the appropriate model. It is easy to see that the MinMax algorithm in Definition 13, as well as our topological characterization, also applies unchanged for ⋄LL.

**Fig. 5:**
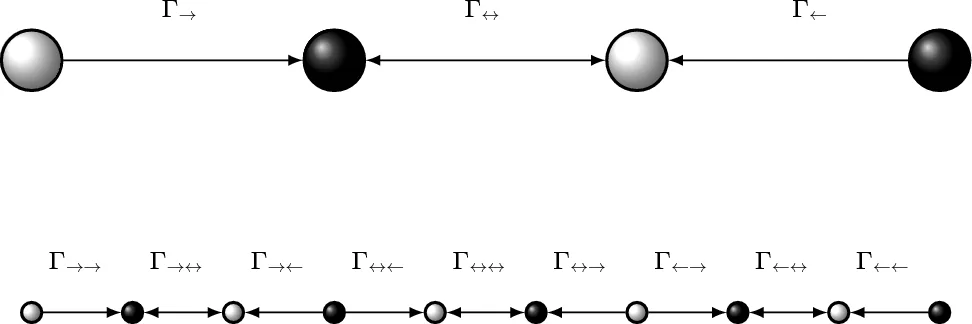
Prefix order for the 1-prefixes and 2-prefixes in the LL model.

In order to prepare the grounds for dealing with the DLL model in Section 7.2.4, we briefly introduce some additional facts about the LL model for $\mathcal {V}=\{0,1\}$, see [27, 42] for details. Most importantly, it is possible to totally order the *k*-prefixes of all admissible graph sequences such that consecutive prefixes are indistinguishable for one of the processes in $\Pi =\{l,r\}$, provided all executions start with the same input assignment. Fig. 5 illustrates this for k=1 and k=2, where the white (resp. black) nodes represent process *l* (resp. *r*).

For example, the interval labeled with the sets of communication patterns $$\Gamma _\rightarrow $$ (resp. $$\Gamma _\leftrightarrow $$) in the top part represent all communication patterns that start with the graph → (resp. ↔) in round 1. Note carefully that any two consecutive σ and $\sigma^{\prime }$ are indistinguishable for the process *p* in-between in executions that start from the same input assignment. Overall, this imposes the order $$\rightarrow \;< \; \leftrightarrow \; < \; \leftarrow $$, which are also used for ordering the sets $$\Gamma _\rightarrow< \Gamma _\leftrightarrow < \Gamma _\leftarrow $$. By construction, every execution $$\gamma _1$$ with communication pattern $$\mathcal {G}_1 \in \Gamma _\sigma $$ and any execution $$\gamma _2$$ with $$\mathcal {G}_2 \in \Gamma _{\sigma '}$$ starting from the same initial configuration $$I(\gamma _1)=I(\gamma _2)$$ satisfy $$d_{\textrm{nu}}(\gamma _1,\gamma _2) \le d_p(\gamma _1,\gamma _2) < 1/2$$ in the *p*-view topology. (Clearly, this holds for both *l* and *r* if $$\mathcal {G}_1, \mathcal {G}_2 \in \Gamma _\sigma $$ as well.)

The bottom part of our illustration is obtained by applying the ordering for k=1 to every edge in the top part. For example, $\Gamma _{\rightarrow \rightarrow }$ (resp. $\Gamma _{\rightarrow \leftrightarrow }$) represents all communication patterns that start with the 2-prefix $\rightarrow \rightarrow$ (resp. $\rightarrow \leftrightarrow$) sharing the same 1-prefix →. Again, $\rightarrow \rightarrow \;< \; \rightarrow \leftrightarrow \;< \; \dots \; < \; \leftarrow \leftarrow$, as well as the sets $\Gamma _{\rightarrow \rightarrow }< \Gamma _{\rightarrow \leftrightarrow }< \dots < \Gamma _{\leftarrow \leftarrow }$, are totally ordered, and any two executions $\gamma _1$ and $\gamma _2$ from direct successors $\Gamma _\sigma <\Gamma _{\sigma '}$ satisfy $d_{\textrm{nu}}(\gamma _1,\gamma _2) \le d_p(\gamma _1,\gamma _2) < 1/4$ for some $p \in \Pi$.

**Fig. 6:**
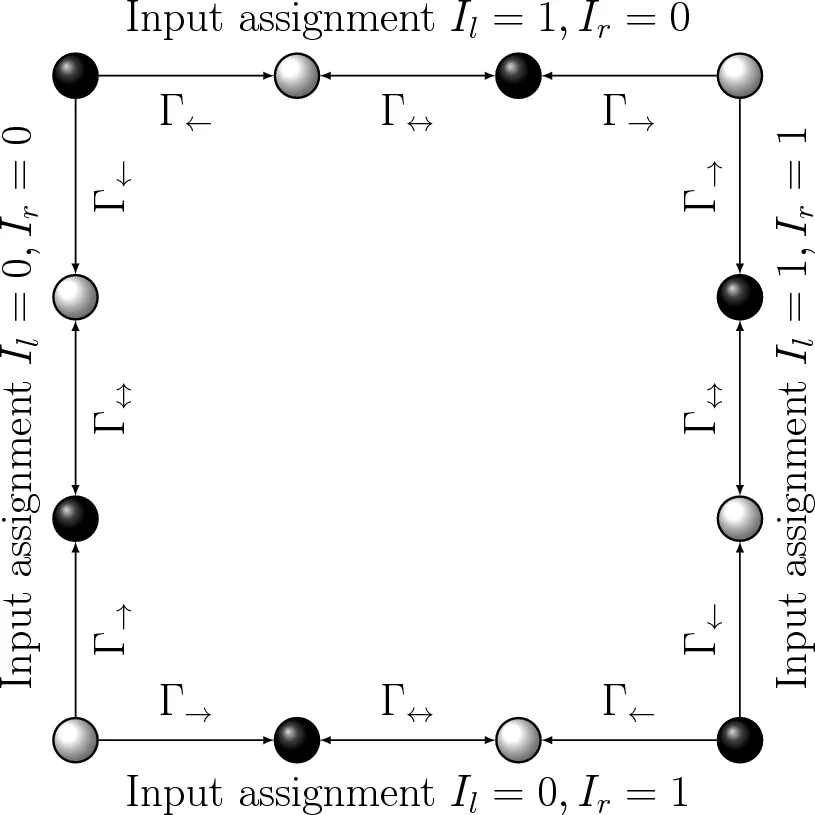
Complete representation of 1-prefixes in the LL model, for all input assignments.

For $\mathcal {V}=\{0,1\}$, four instances of these communication patterns, each representing one of the 4 different possible input assignments, are connected in a cycle, as shown in Fig. 6.^3^ Thanks to this arrangement, the argument that any two executions $\gamma _1$ and $\gamma _2$ from direct successors $\Gamma _\sigma <\Gamma _{\sigma '}$ satisfy $d_{\textrm{nu}}(\gamma _1,\gamma _2) \le d_p(\gamma _1,\gamma _2) < 1/4$ for some $p \in \Pi$ also extends to $\gamma _1$, $\gamma _2$ that are indistinguishable for a corner node. This is particularly relevant for the strong decision sets $\Sigma _0$ and $\Sigma _1$ induced by the MinMax algorithm: According to our considerations above, there are only two boundary points here, namely, the execution $\gamma _r \in \Sigma _1$ with $I_l(\gamma _r)=0$, $I_r(\gamma _r)=1$ and $\mathcal {G}= \{\leftarrow \}^\omega$ (corresponding to the bottom-right black node in Fig. 6), and $\gamma _l \in \Sigma _1$ with $I_l(\gamma _l)=1$, $I_r(\gamma _l)=0$ and $\mathcal {G}= \{\rightarrow \}^\omega$ (the top-right white node in Fig. 6). $\Sigma _1$ comprises all executions corresponding to the right edge in our figure, $\Sigma _0$ is made up by the executions corresponding to all the other edges.

It is instructive to relate the situation to the problem of solving terminating consensus in sub-models of LL. According to Corollary 4.10, one has to remove at least two fair or two pairs of unfair executions (see Definition 4.9) from the connected space of admissible LL executions Σ for this purpose. In particular, just excluding the above boundary executions $\gamma _l$ and $\gamma _r$ from Σ allows to solve terminating consensus: If e.g. $\{\leftarrow \}^\omega$ cannot occur, *l* only needs to wait until the (guaranteed) message from *r* arrives and to decide on *r*’s input value then, whereas *r* can decide on its own input already initially.

Topologically, this exclusion partitions the connected LL space Σ (which is homeomorphic to a 1-sphere in the case of $$\mathcal {V}=\{0,1\}$$, see Fig. 6), into at least two connected (and hence clopen) components: Since the decision function of terminating consensus is continuous, this is mandatory for mapping the resulting space $\Sigma^{\prime }$ to the discrete and hence disconnected space $$\mathcal {V}$$. For stabilizing consensus, a separation into *clopen* sets is not needed: disjoint sets with included boundary points in between suffice here, since semi-continuous decision functions can map such sets to a discrete space, recall Fig. 2 and Fig. 1.

#### Empty kernel impossibility

Among the few stabilizing consensus impossibility results known so far is [5, Thm. 6], which states that any infinite graph sequence with an empty kernel that is admissible for a message adversary makes the problem unsolvable. Informally, the kernel of a communication pattern $\mathcal {G}$ is the set of processes that can reach all other processes (typically via multiple hops) infinitely often, i.e., are broadcasters in every suffix of $\mathcal {G}$.

In the case of an empty kernel, there is a round $t_0$ from which on no process reaches all other processes in Π: Every message broadcast by process *p* in any round $t\ge t_0$ will fail to reach some recipient $q \in \Pi$. Now consider the sub-model where all processes start only at round $t_0$, which boils down to executions γ that have a communication pattern $\mathcal {G}$ that starts with a prefix of $t_0$ empty graphs and $bc(\gamma )=\emptyset$. Corollary 6.7 asserts that stabilizing consensus is impossible here.

#### Bounded rootedness possibility

In [5], Charron-Bost and Moran also introduced a generalized version of the simple MinMax algorithm stated in Definition 13, which they called safe MinMax. Informally, it allows the processes to take (i) the maximum over a suffix of (ii) the minumum input value over a prefix, which both get longer and longer with increasing rounds. In our setting, it can be expressed via the following decision function:

14 $$\begin{aligned} \Delta _p(C^t) = \max _{q \in {{\,\textrm{In}\,}}_p(\theta _p(t)+1,t)} \left\{ \min _{s \in HO_q(C^{\theta _p(t)})} I_s(\gamma ) \right\} . \end{aligned}$$

Herein, $\theta _p(t)$ is a *cut-off function*, computable by process *p*, which must satisfy $\lim _{t\rightarrow \infty }\theta _p(t)=\lim _{t\rightarrow \infty } (t-\theta _p(t)) = \infty$. The set ${{\,\textrm{In}\,}}_p(\theta _p(t)+1,t)$ denotes the processes *q* from which *p* has heared of by round *t* when only considering messages that have been sent by *q* in or after round $\theta _p(t)+1$.

It was proved that safe MinMax allows to solve stabilizing consensus for every message adversary that satisfies bounded rootedness, which implies that, in every execution γ, there is some broadcasting time $T \in \mathbb {N}$ (possibly unknown to the processes) and a non-empty set of broadcasters, which reach every process by round t+T when broadcasting in round t≥1.

Again, it is easy to show that Definition 14 leads to the strong decision sets already used in Section 7.1. The argument is essentially the same, except that a more complex condition regarding when the smaller input value w<v is received by other processes in $\delta _i'\in \Sigma _w$ occurring in the sequence $\delta _1',\delta _2', \dots \rightarrow \gamma \in \Sigma _v$ needs to be used. More specifically, consider any round $t_i$ where $\theta _q(t_i)$ is large enough such that *every* process *q* has already heared of at least once from every process it will ever hear of in $\delta _i'$, but small enough such that *q* hears, by round $t_i$, about a message sent by at least one process $p'\in bc(\delta _i')$ (by assumption with $I_{p'}(\delta _i')=w$) after round $\theta _q(t_i)$. According to [5, Lem. 7], this guarantees that $\Delta _q(t_i)=w$. Since the broadcasting time *T* is fixed and $\lim _{t\rightarrow \infty }\theta _q(t)=\lim _{t\rightarrow \infty } (t-\theta _q(t)) = \infty$, the latter can be guaranteed for any round $t\ge t_i$ as well. For our desired sequence $\delta _1',\delta _2', \dots \rightarrow \gamma \in \Sigma _v$, choosing any non-decreasing sequence $t_i$ with $\lim _{i\rightarrow \infty } t_i=\infty$ will do the job.

For the limit sequence γ, this obviously implies that no process *q* ever hears from $p^{\prime }$, so $$p'\not \in bc(\gamma )$$ as needed. As in the argument already used in Section 7.1, we can hence choose a corresponding sequence $$\delta _1,\delta _2, \dots \rightarrow \gamma \in \Sigma _v$$ that is identical to $$\delta _1',\delta _2', \dots $$, except that $p^{\prime }$ also has the initial value $$I_{p'}(\delta _i)=v > w$$. Since it also converges to γ, this again proves that $$\gamma \in {{\,\mathrm{\partial in}\,}}\Sigma _v$$ is a limit point.

#### Delayed lossy-link model impossibility

The safe MinMax algorithm given in Definition 14 in Section 7.2.3 also allows to solve stabilizing consensus under the *bounded delayed lossy-link* message adversary BDLL defined as $\bigcup _{T\ge 0} \bigl ( \bigcup _{k=0}^T \{\text {---}\}^k LL\bigr )^\omega$, which allows one round of the classic LL model ($LL=\{\leftarrow ,\leftrightarrow ,\rightarrow \}$) to be interleaved with silence periods (with no communication at all) of at most *T* rounds, for some unknown *T*, in every execution.

A natural question is whether restricting the maximum duration of the silence periods in any execution to some fixed *T* is mandatory for solving stabilizing consensus. Since the LL model guarantees a non-empty kernel in every execution, which carries over to BDLL, this question is related to the more general question posed in [5]: Is a non-empty kernel in every execution, which is known to be necessary (recall Section 7.2.2) for solving stabilizing consensus, also sufficient? Clearly, safe MinMax would not work here, as one cannot guarantee that the cut-off function $\theta _p(t)$ properly covers arbitrarily finite silence periods, but there might be other protocols.

In [23], Felber and Rincon Galeana answered this question negatively: They introduced the *delayed lossy-link* message adversary DLL defined as $\bigl (\{\text {---}\}^*LL\bigr )^\omega$, which allows arbitrary but finite silence periods in every execution, and showed that it is impossible to solve stabilizing consensus in this model. We will use our topological characterization for providing an alternative proof of this fact.

Restricting our attention to binary stabilizing consensus, i.e., $\mathcal {V}=\{0,1\}$, we assume for a contradiction that there is a correct stabilizing consensus protocol $\mathcal {A}$ for the DLL model. Let $\Sigma _0$ and $\Sigma _1$ be the resulting decision sets, which must be semi-open according to Theorem 5.7. We will construct an admissible execution, which cannot be assigned to any decision set, which provides the required contradiction.

**Fig. 7:**
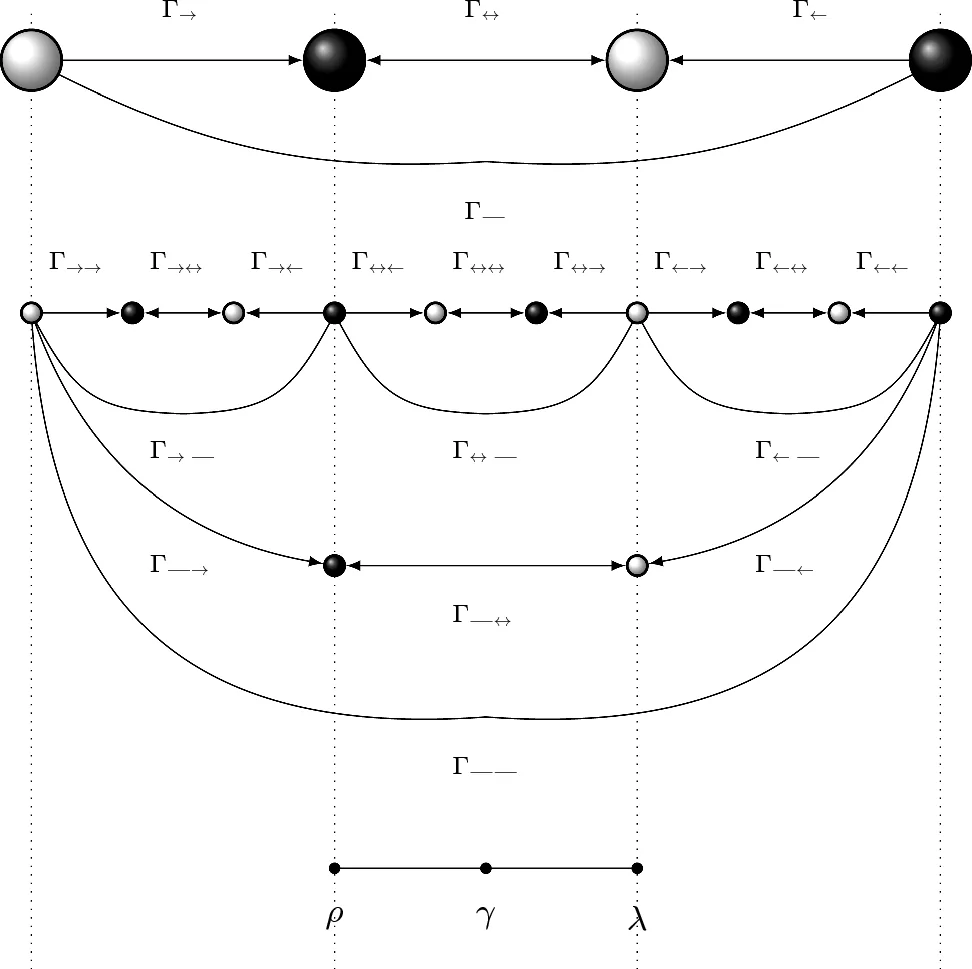
Representation of all 1-prefixes and 2-prefixes in the DLL model, for a fixed input assignment.

We start with using our stabilizing consensus protocol $\mathcal {A}$ in the LL model, which is of course a sub-model of DLL: Fig. 5 indeed contains all the graph sequences in Fig. 7. Let $\hat{\Sigma }_0\subseteq \Sigma _0$ and $\hat{\Sigma }_1 \subseteq \Sigma _1$ be the corresponding decision sets. From our topological considerations at the end of Section 7.2.1, where we elaborated on the limit points that connect $\Sigma _0$ and $\Sigma _1$, we know that there must be some boundary point γ, w.l.o.g. $\gamma \in {{\,\mathrm{\partial in}\,}}\hat{\Sigma }_0$, which is also a limit point of $\hat{\Sigma }_1$. Note that γ is hence also a limit point in the boundary of the DLL decision sets $\Sigma _0$ and $\Sigma _1$.

Unfortunately, however, all that we know about $\mathcal {A}$ is that it produces semi-open decision sets. In particular, we do not know anything about the set of boundary points $\mathcal {A}$ produces, besides that ${{\,\mathrm{\partial in}\,}}\hat{\Sigma }_v$ and $\hat{Y}=\bigcup _{v\in \mathcal {V}} {{\,\mathrm{\partial in}\,}}\hat{\Sigma }_v$ are nowhere dense by Lemma 5.4. Therefore, we cannot just assume that there is some boundary point γ that squarely separates $\hat{\Sigma }_0$ and $\hat{\Sigma }_1$, but need a slightly more refined approach.

We start with some definitions: For an arbitray execution γ with graph sequence $\mathcal {G}$, let $\sigma =\mathcal {G}|_{k+1}$ be the (k+1)-prefix of $\mathcal {G}$, for any k≥0. Recall that, for any execution β with graph sequence $\mathcal {B}$ starting from the same initial configuration $I(\gamma )=I(\beta )$, $\mathcal {B}|_{k+1}=\mathcal {G}|_{k+1}$ guarantees $\beta |_{k+1} \sim _{l,r} \gamma |_{k+1}$. Let λ and ρ be the (unfair, recall Definition 4.9) admissible executions based on the graph sequence $\sigma \{\rightarrow \}^\omega$ and $\sigma \{\leftarrow \}^\omega$, respectively. We call them *LL border executions*, since, for every prefix size k+1+ℓ, $\ell \ge 0$, the sets of communication patterns $\hat{\Sigma }_{\sigma \{\rightarrow \}^\ell }$ (resp. $\hat{\Sigma }_{\sigma \{\leftarrow \}^\ell }$) starting with $\sigma \{\rightarrow \}^\ell$ (resp. with $\sigma \{\leftarrow \}^\ell$) are the smallest (resp. largest) element in the corresponding prefix-order, as illustrated for k=1 and k=2 in Fig. 5. By construction, both $\lambda , \rho \in B_{2^{-(k+1)}}(\gamma )$, where $B_{2^{-(k+1)}}(\gamma )$ denotes the open ball with radius $2^{-(k+1)}$ around γ for the distance function $d_{\textrm{nu}}$.

It follows from the validity property (V) that the right and left corner executions $\gamma _r$ (resp. $\gamma _l$) on the bottom edge in Fig. 6, which are based on the graph sequences $\mathcal {G}_r=(\leftarrow )^\omega$ (resp. $\mathcal {G}_l=(\rightarrow )^\omega$), satisfy $\gamma _r \in \hat{\Sigma }_1$ and $\gamma _l\in \hat{\Sigma }_0$. In every prefix-order, i.e., for arbitrary (k+1)-prefixes, k≥0, there must hence be at least one prefix σ, of some execution γ, such that the corresponding LL border executions λ, ρ satisfy $\lambda \in \hat{\Sigma }_0$ and $\rho \in \hat{\Sigma }_1$. After all, somewhere, the decision value must flip from 0 to 1 when going forward in the prefix-order. Again, this must also hold true in the DLL model, i.e., $\lambda \in \Sigma _0$ and $\rho \in \Sigma _1$, see Fig. 7 for an illustration for k=1.

For any m≥0, we now define the admissible parametrized DLL execution λ(m), which is based on the graph sequence $\sigma \{\text {---}\}^m\{\rightarrow \}^\omega$, and ρ(m), which is based on $\sigma \{\text {---}\}^m\{\leftarrow \}^\omega$. Besides $\lambda (0)=\lambda$ and $\rho (0)=\rho$, they satisfy

15 $$\begin{aligned} d_l(\lambda (m),\lambda )&=0,\end{aligned}$$

16 $$\begin{aligned} d_r(\rho (m),\rho )&=0. \end{aligned}$$

Moreover, since λ(m) and ρ(m) start from the same prefix σ,

17 $$\begin{aligned} \lambda (m)|_{k+m} \sim _{l,r} \rho (m)|_{k+m}. \end{aligned}$$

Due to Eq. (17), we find that, for any m≥0, the respective output values after round k+m satisfy

18 $$\begin{aligned} O_l\bigl (\lambda (m)|_{k+m}\bigr )&=O_l\bigl (\rho (m)|_{k+m}\bigr ),\end{aligned}$$

19 $$\begin{aligned} O_r\bigl (\lambda (m)|_{k+m}\bigr )&=O_r\bigl (\rho (m)|_{k+m}\bigr ). \end{aligned}$$

However, since $\lambda \in \Sigma _0$ and $d_l(\lambda ,\lambda (m))=0$ by Eq. (15), there is a round $k_l\ge k$ after which $O_l=0$ in both λ and λ(m). Analogously, since $\rho \in \Sigma _1$, there is a round $k_r\ge k$ after which $O_r=1$ in both ρ and ρ(m). For $m \ge \max \{k_l,k_r\}-k$, Eq. (18) (resp. Eq. (19)) ensures that $O_l=0$ also in ρ(m) (resp. $O_r=1$) also in λ(m). Consequently, the Stabilizing Agreement property (SA) is violated both in λ(m) and ρ(m) in round k+m, so the executions cannot have stabilized by round k+m. In the notation of [23], any such execution has a *conflicting* prefix. Since both k≥0 and $m \ge \max \{k_l,k_r\}-k$ can be chosen arbitrarily large, there must be at least one non-stabilizing execution γ that cannot be uniquely assigned to either $\Sigma _0$ or $\Sigma _1$; the stipulated stabilizing consensus protocol $\mathcal {A}$ hence cannot be correct.

#### One-message lossy link possibility

As our final example for demonstrating the explanatory power of our topological approach, we consider some apparently minor strengthening of the DLL model, which nevertheless makes stabilizing consensus trivially solvable in the model where both processes are active from $t_l^a=t_r^a=0$ on. We call it the *one-message lossy link* model, where the set of allowed graph sequences is the union of $\bigl \{\{\text {---}\}^*\rightarrow \{\text {---}\}^\omega \bigr \}$ and $\bigl \{\{\text {---}\}^*\leftarrow \{\text {---}\}^\omega \bigr \}$. Obviously, in any admissible execution, exactly one message (either → or ←) is successfully received.

Despite the infinite suffix $\{\text {---}\}^\omega$, it is trivial to solve stabilizing consensus in this model: Initially, *l* and *r* set $O_l=I_l$ and $O_r=I_r$ and keep that value, unless on of them, $x \in \{l,r\}$, receives a message containing the other’s current output value *O*. If so, *x* sets $O_x=O$ and keeps that value. By contrast, it will turn out that terminating consensus cannot be solved in this model.

Our one-message lossy link model is hence arguably the simplest conceivable model for demonstrating the solvability border between terminating consensus and stabilizing consensus.

Our topological characterization easily reveals what happens here. It suffices to characterize the decision sets $\Sigma _0$ and $\Sigma _1$ imposed by this protocol, in the restricted setting where $I_l=0$ and $I_r=1$ are the same in all admissible executions; therefore, we can consider admissible executions and graph sequences as being equivalent. Generalizing our considerations to proper stabilizing consensus protocols is straightforward.

For i≥0, let $\eta =\{\text {---}\}^\omega$, $\alpha _i = \{\text {---}\}^i\rightarrow \{\text {---}\}^\omega$, and $\beta _i=\{\text {---}\}^i\leftarrow \{\text {---}\}^\omega$. Then, $\Sigma _0=\{\alpha _i\mid i \ge 1\}$ and $\Sigma _1=\{\beta _i\mid i \ge 1\}$. Moreover, for any $i\ge 1, k\ge 1$, we find $d_l(\alpha _i,\alpha _k)=0$ and $d_l(\alpha _i,\eta )=0$ (since *l* never gets any message in any of these executions), as well as $d_r(\beta _i,\beta _k)=0$ and $d_r(\beta _i,\eta )=0$. On the other hand, $d_r(\alpha _i,\alpha _{i+k})=2^{-(i+1)}$ and $d_r(\alpha _i,\eta )=2^{-(i+1)}$, as well as $d_r(\beta _i,\beta _{i+k})=2^{-(i+1)}$ and $d_l(\beta _i,\eta )=2^{-(i+1)}$. Since the triangle inequality reveals $d_l(\alpha _i,\beta _k) \le d_l(\alpha _i,\eta ) + d_l(\eta ,\beta _k) = d_l(\eta ,\beta _k)=2^{-(k+1)}$ and $d_r(\alpha _i,\beta _k) \le d_r(\alpha _i,\eta ) + d_r(\eta ,\beta _k) = d_r(\alpha _i,\eta )=2^{-(i+1)}$, it turns out that any $\alpha _i$ is a limit point of the sequence $\beta _1,\beta _2, \dots$ in the *l*-view topology. In addition, trivially, any $\alpha _i$ is also a limit point of $\alpha _1,\alpha _2, \dots$ in the *l*-view topology. Analogously, any $\beta _i$ is a limit point of both the sequence $\alpha _1,\alpha _2, \dots$ and $\beta _1,\beta _2,\dots$ in the *r*-view topology. Therefore, Corollary 4.8 implies that terminating consensus is impossible in this model.

As a consequence, in the non-uniform topology, the diameter (see Definition 6.5) of both $\Sigma _0$ and $\Sigma _1$ is 0. Moreover, ${{\,\mathrm{\partial in}\,}}\Sigma _0 = \Sigma _0$ and ${{\,\mathrm{\partial in}\,}}\Sigma _1 = \Sigma _1$, whereas ${{\,\mathrm{\partial }\,}}{{\,\mathrm{\partial in}\,}}\Sigma _0 = \Sigma _1$ and ${{\,\mathrm{\partial }\,}}{{\,\mathrm{\partial in}\,}}\Sigma _1 = \Sigma _0$ since both $\Sigma _0$ and $\Sigma _1$ are dense in Σ, i.e., $\overline{\Sigma }_0 = \overline{\Sigma }_1 = \Sigma$. It follows that the conditions for Theorem 5.7 are satisfied, such that stabilizing consensus is indeed solvable in this model. Note that case (c) in the proof of Theorem 5.7, i.e., Eq. (10), cannot occur here at all, since ${{\,\mathrm{\partial }\,}}{{\,\mathrm{\partial in}\,}}\Sigma _0 \cap {{\,\mathrm{\partial }\,}}{{\,\mathrm{\partial in}\,}}\Sigma _1 = \emptyset$.

## Conclusions

We provided a complete characterization of the solvability/impossibility of deterministic stabilizing consensus in any computing model with benign process and communication faults using point-set topology. Using the topologies for infinite executions introduced in [32] for terminating consensus, we proved that semi-open decision sets and semi-continuous decision functions as introduced in [34] are the appropriate means for this characterization. We also showed that multi-valued stabilizing consensus with weak and strong validity are equivalent, like for terminating consensus. We demonstrated the power of our topological characterization by easily applying it to (variants of) all the known possibility and impossibility results for stabilizing consensus known so far.

## Data Availability

No datasets were generated or analysed during the current study.

## Footnotes

1 Since we are not aware of any earlier usage of Levine’s results in the distributed computing context, our findings constitute another instance of the utility of purely theoretical (here: mathematical) research for eventually advancing science in more applied areas.

2 Whereas our topological modeling would also allow byzantine processes, we deliberately restricted our attention to benign ones, where processes can at most commit send and/or receive omissions or crashes: Byzantine processes would require different validity conditions, the detailed treatment of which would blow up our paper considerably, without adding anything substantially new.

3 Readers familiar with combinatorial topology in distributed computing, see e.g. [43–46], will immediately recognize that this is just the protocol complex arising after 1 round of execution, which is just a graph since we only have n=2 processes.
